# Premenstrual syndrome: new insights into etiology and review of treatment methods

**DOI:** 10.3389/fpsyt.2024.1363875

**Published:** 2024-04-23

**Authors:** Stefan Modzelewski, Aleksandra Oracz, Xawery Żukow, Kamila Iłendo, Zofia Śledzikowka, Napoleon Waszkiewicz

**Affiliations:** Department of Psychiatry, Medical University of Bialystok, Białystok, Poland

**Keywords:** PMS, PMDD, SSRI, allopregnanolone, treatment

## Abstract

Premenstrual syndrome (PMS) is a common disorder affecting women of reproductive age, with an estimated global prevalence of 47.8%, with severe symptoms occurring in 3-8%, significantly affecting daily functioning. GABA conductance and changes in neurosteroid levels, particularly allopregnanolone, are suspected to play a substantial role in the disorder’s etiology. In this paper, we provide an overview of recent reports on the etiology and recognized therapeutic approaches, encompassing both pharmacological and non-pharmacological interventions. Our examination includes studies on SSRIs, hormonal agents, neurosteroids, supplementation, and therapeutic roles. We aim to determine the most favorable treatment regimen by comparing medication effects and alternative methods. The treatment of PMS is crucial for enhancing the quality of life for affected women. Medications used in PMS treatment should be individually selected to achieve the best therapeutic effect, considering the clinical situation of the patients.

## 1 Introduction

Many women of reproductive age experience dysphoria and physical symptoms approximately two weeks before menstruation (1). The mentioned discomfort, both physical and psychological, associated with the luteal phase of the menstrual cycle and typically resolving when menstruation ends, is defined as premenstrual syndrome (PMS) (2). The global prevalence of premenstrual syndrome is estimated at 47.8% (3), while the most severe form of PMS - premenstrual dysphoric disorder (PMDD) affects 3-8% of women of reproductive age (4). What is more, the PMDD is classified as a gynecological diagnosis in the ICD-11 classification and as a psychiatric diagnosis in the Diagnostic and Statistical Manual of Mental Disorders (DSM-5) (5). That indicates the complexity of the disorder and is a reminder of the widespread spectrum of symptoms. The most common mental symptoms of PMS include irritability, tearfulness, anxiety, and depressed mood. Physical ones, on the other hand, mainly involve abdominal bloating, breast tenderness, and headaches (6). Hormonal changes, stress, diet, and alterations in neurotransmission are considered the most significant risk factors (7). It is also suspected that the severity of PMS is higher in unmarried women compared to married women, those with lower economic status, and those with a family history of similar cases (8). Behavioral and social factors also play a role, including medication use (including contraceptives), smoking, alcohol and caffeine consumption, and even education. Age, past pregnancies, and previous menstrual history have also been evaluated, but there is still no complete consensus on how they impact the development of the disorder (9). Diagnosing premenstrual syndrome is possible only after ruling out other conditions that could better explain the experienced discomforts (10). The treatment primarily focuses on alleviating symptoms, and we will delve into this aspect further in our discussion.

## 2 Etiopathogenesis

The pathogenesis of PMS is intricate and not fully understood. Several theories attempt to explain the causes of its symptoms.

**Figure 1:**
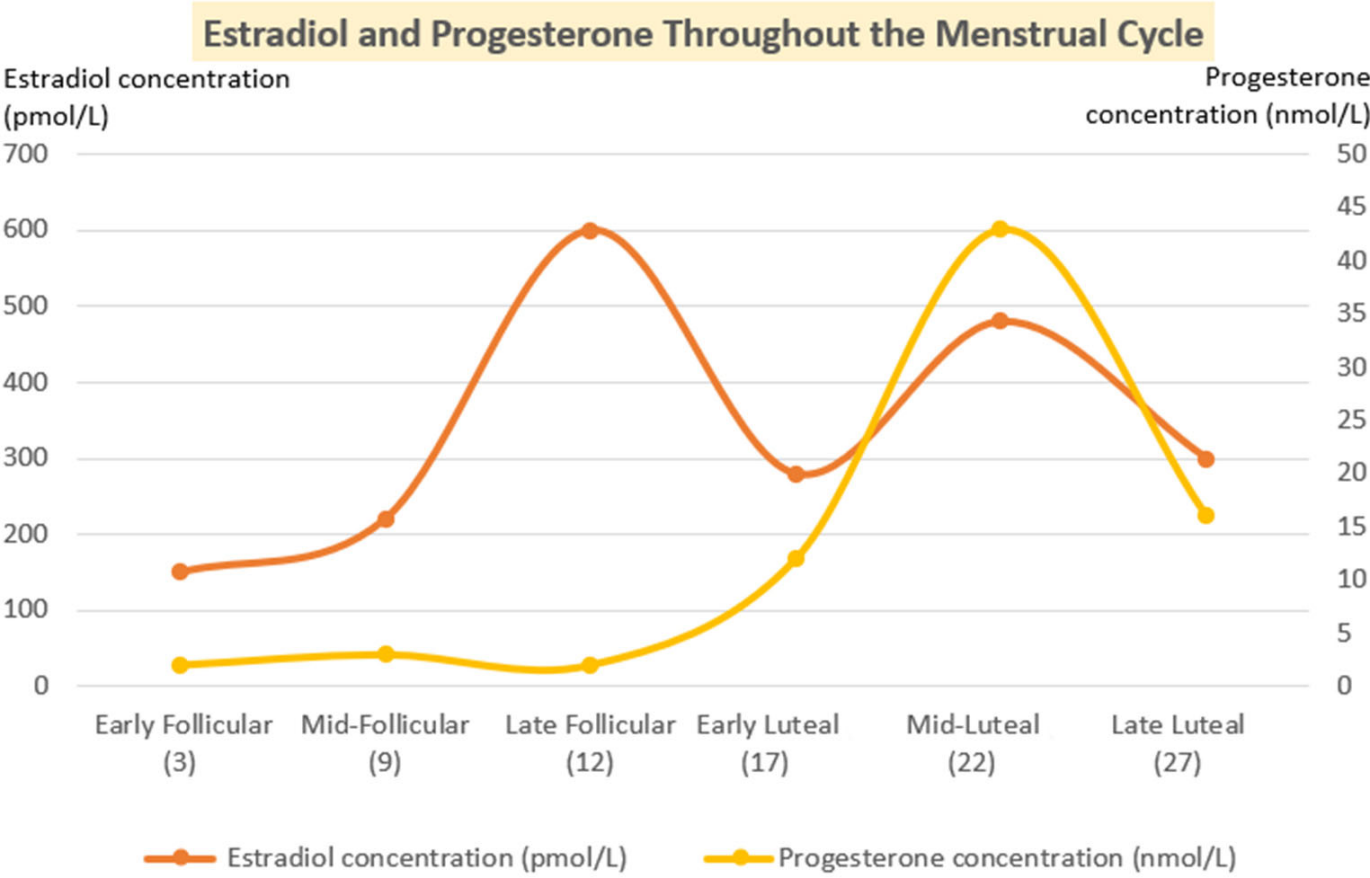
Concentrations of estradiol and progesterone during the menstrual cycle.

Classically, PMS has been linked to hormonal fluctuations during the monthly cycle, with mood deterioration and increased anxiety primarily associated with decreases in estrogen and progesterone (**Figure 1**).

### 2.1 The role of sex steroids and their derivatives - central role for allopregnanolone?

Recently, particular attention has been given to the progesterone metabolite allopregnanolone (11). Allopregnanolone is an allosteric modulator of the GABA receptor in the CNS, which binds to the alpha and beta subunits at residues m1-m3 (12), which explains its broad effects on multiple CNS pathways (13). Moreover, allopregnanolone synthesis can occur *de novo* not only in the brain but also in the ovaries and adrenal glands due to the presence of necessary enzymes in these organs needed for its production (14). Understanding the significance of allopregnanolone in alleviating PMS symptoms may provide crucial information about the cause of the disorder itself. Significantly, when utilized as a novel drug (brexanolone) for postpartum depression (PPD) treatment, it not only mitigates affective disorders (15) but also suppresses the inflammatory response. This dual action could potentially alleviate the severity of peripheral symptoms, including pain (16). The steroidal structure of progesterone and its metabolites enables them to penetrate the blood-brain barrier when formed peripherally, as observed in the ovaries (14). It is important to note that the presence of PMS is a risk factor for PPD (17). Both conditions are believed to be caused by hormonal changes, specifically the increase and subsequent withdrawal of sex hormones (18, 19), and the existence of subgroups of susceptible individuals (20, 21). Due to these associations and the increased interest in neurosteroids, allopregnanolone has become one of the most commonly linked substances to the etiology of PMS in recent years (20).

Women experiencing premenstrual symptoms demonstrate an impaired stress response (22). This may be precisely linked to the action of steroid hormones, which, through various mechanisms, inhibit the activity of the HPA axis, starting at the level of the PVN (23). Progesterone, or more specifically, its metabolite — allopregnanolone, enhances GABA conductance and suppresses CRH formation in hypothalamic cells. In contrast, estrogen inhibits the generation of free radicals, resulting in a reduction of oxidative stress in the body (24). What is more, Granda et al. suggest that abnormal oxidative and inflammatory activity may occur in PMS (25). It is possible that in PMS, there is an abnormal response to estradiol and an increase in oxidative stress, given that antioxidants in high concentrations have a pro-inflammatory effect and estradiol has a second peak concentration in the early luteal phase (26). The significance of estrogen metabolites producing oxygen radicals (27) is noteworthy. However, the current research does not allow for a clear assessment of the role of oxidative stress (28–30). Interestingly, there are no discernible differences in hormone levels during the monthly cycle between healthy women and those suffering from PMS (31). However, concentrations of allopregnanolone and its conversion from progesterone are higher in women with the PMDD (32). This suggests a disturbance in the metabolic pathway of progesterone in women who are affected and implies the existence of a subgroup of women sensitive to hormone concentrations. This sensitivity is supported by the findings of Schmidt et al., who demonstrated that re-administration of progesterone to women suffering from PMS while taking a leuprolide resulted in a recurrence of symptoms (33).

Furthermore, women with PMS, after blocking 5-alpha-reductase, a crucial enzyme for allopregnanolone production, experienced significantly reduced premenstrual symptoms (34). In contrast, during the follicular phase, women with PMDD who took allopregnanolone as part of another study showed reduced GABA-A receptor sensitivity (35). These data underscore the crucial role of this metabolite in the described disorder: High allopregnanolone levels may explain why the stress response in women with premenstrual disorders is blunted (36), given the mentioned above impact of GABA conductance on CRH.

However, as explained above, blocking its synthesis provides relief to patients. The explanation for this situation may lie in the reaction to substances in the CNS itself. Due to their structure, steroid hormones can interact not only trans-membrane to the cell but also through the G-protein-bound receptor, leading to changes in the cell genome (37). Theoretically, with an increase in progesterone, there is an unimodal increase in allopregnanolone, and an adaptation - a down-regulation of the receptor to maintain constant inhibition of GABA (14). With a decrease in the concentration of the substance in the later luteal phase, the physiological GABA-glutamate balance could be disturbed: Adaptive changes do not keep up with the contraction in allopregnanolone, which was higher at baseline in affected women, and GABA receptors are not restored in time, leading to impaired GABA conductance. This could explain, among other things, the increased activity of the prefrontal cortex (38), as observed in imaging studies.

**Figure 2:**
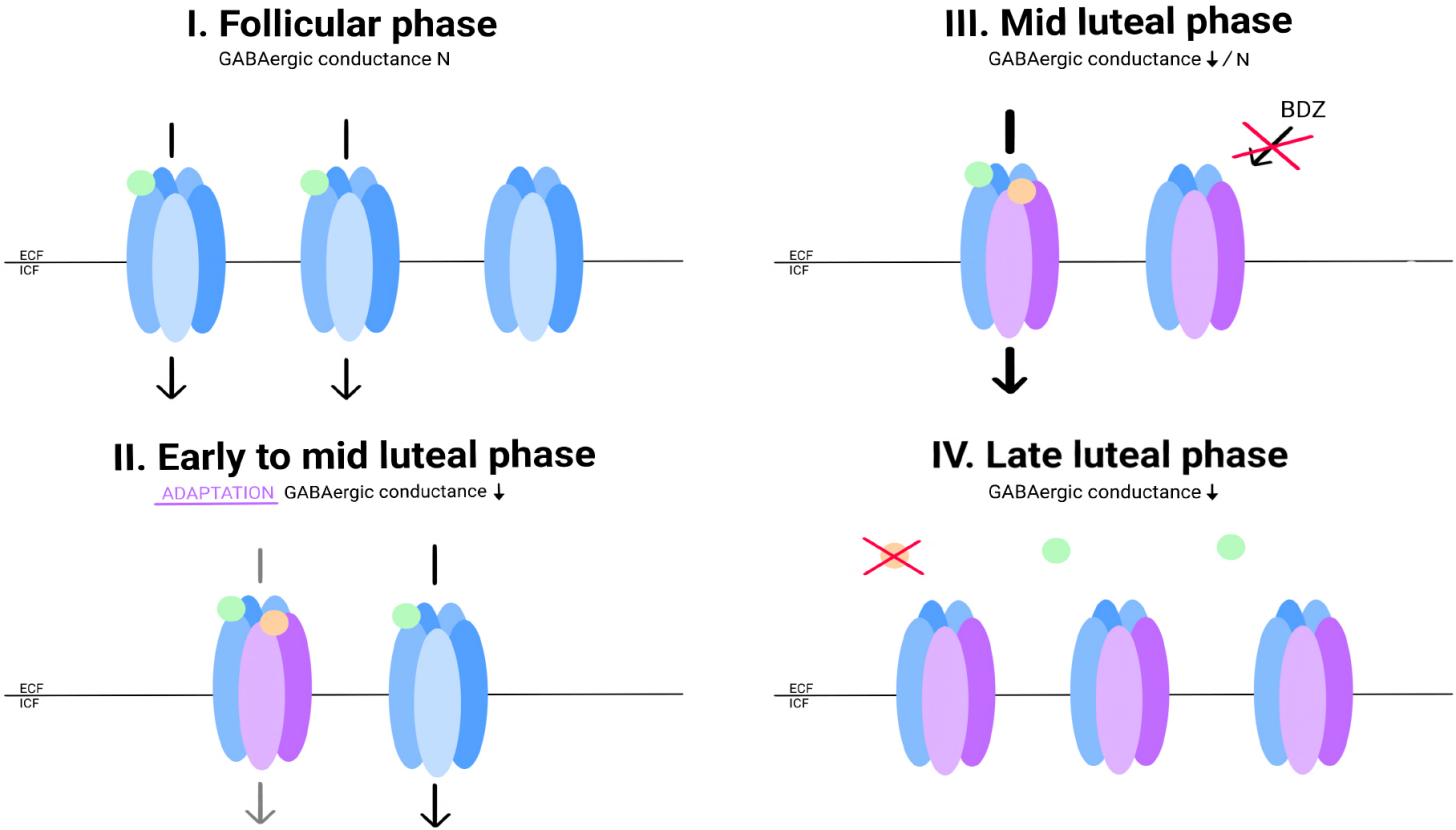
Allopregnanolone concentrations and hypothetical changes in GABA conductance in PMS patients. During the follicular phase, progesterone and allopregnanolone concentrations are low. Expression of selected GABA receptor subunits is not increased. GABA conductance functions properly (I). With increasing concentrations of allopregnanolone, the conformation of the GABA-A receptor is affected: the expression of the alpha4 subunit, and probably delta, is increased. In a group of women with PMS, there is a paradoxical decrease in GABA conductance under the influence of allopregnanolone (II). This condition explains the paradoxical effect of flumenazil in women with PPMD. The GABA-A receptor in this conformation is insensitive to BZDs. At the highest concentration of allopregnanolone in the cycle, GABA-A conductance is mainly regulated by it (III). Allopregnanolone does not reach high enough concentrations in the cycle to induce the expected allosteric modulator effect. Its concentration begins to fall, forcing readaptations within the GABA-A receptor. Until the conformation of the molecules returns to ''physiological'', inhibition may be impaired (IV). - Gamma-aminobutyric acid (GABA); - Allopregnanolone; - GABA receptor; - GABA receptor with altered subunit expression in response to allopregnanolone.

According to this assumption, it would not be the neurosteroid concentration itself that causes the onset of symptoms, but rather the decrease in concentration. The GABA-A receptor appears to adapt to neurosteroid concentrations through changes in its conformation (39). Women with PMDD have lower sensitivity to benzodiazepines, as well as pregnanolone, which may be related to receptor adaptation involving increased expression of the delta subunit. This subunit is insensitive to benzodiazepines but highly sensitive to allopregnanolone (40). Its increased expression, along with the other subunits, may reflect an attempt to adapt to falling allopregnanolone concentrations at the end of the luteal phase, especially since a study by Timby et al. indicates that women with PMDD have altered sensitivity to allopregnanolone (35). A similar theory regarding PPD was presented by Maguire et al. (41). In the case of the monthly cycle, it appears that the distinction lies not so much in the concept of abnormal adaptations taking place but in the severity of their proportions. The long-drawn modulation in PPD throughout the 3rd and 4th trimesters of pregnancy may be linked to both the effects of prolonged exposure of GABA cells to allopregnanolone, leading to adaptations at the level of the receptor and cell genome, and significantly higher progesterone concentrations than in the luteal phase (42). In contrast, changes in PMS may occur only through a pathway of rapid adaptation to allopregnanolone involving structural changes in the GABA A receptor, which would explain the lower severity of symptoms. Additionally, the relationship between the two pathologies is indicated by the fact that PMS predisposes to PPD (17), and similar to PPD, in PMS, we observe a subgroup of women sensitive to hormonal fluctuations (21) (**Figure 2**).

However, it is important to remain skeptical when discussing the connection between the onset of PMS and fluctuations in progesterone derivative levels. The studies by Schmidt et al. (33, 43) showed that eliminating hormone fluctuations during the luteal phase is not enough to prevent the onset of PMS symptoms. What is interesting, the researchers found that there was a subgroup that was sensitive to hormonal fluctuations: only patients with a history of PMS responded to hormonal interventions compared to a group of healthy women. Based on these findings and considering the abnormal response to BDZ in PMS patients, it can be concluded that abnormal adaptive responses of the GABA A receptor are one of the main, but not the only, problems faced by women with PMS. Furthermore, Schmid et al. also indicated in their more recent study that it is not high progesterone levels sustained over a long period, but changes in progesterone concentration that are key in triggering symptoms. This study provides a more complete understanding of the role of sex hormones in the disorder - the findings indicate that it is not re-administration but changes in sex hormone concentration that may be crucial. In both cases, this may indicate abnormal adaptations of the GABA A receptor (44).

The additional importance of hormones is underscored by estrogen’s ability to promote growth factors, such as BDNF (22, 45). SSRIs, used in the treatment of PMS, also stimulate its formation, and their effectiveness in treatment serves as indirect evidence of the importance of disturbances in serotonergic conduction in the etiopathogenesis of this pathology (46). Imaging studies further provide evidence of altered GABA and serotonergic conduction in the amygdaloid nucleus and prefrontal cortex in patients affected by PMDD (47).

### 2.2 Rapid action of SSRIs in PMS - potential mechanism

Due to the rapid response to treatment with SSRI drugs, a different mechanism should be considered compared to the classical model found in affective disorders (48). In the classical model, the drugs take effect after about 3 weeks, while in the case of PMS, no such time gap is observed. A strong argument for the importance of serotonergic conduction is the lower peripheral blood serotonin levels during the luteal phase in women with PMS (49, 50). Use of drugs from SSRI group, leads to an increase in serotonin concentration in the synaptic cleft. An increase in serotonergic neurotransmission is the result (51). Furthermore, recent studies have shown that during the monthly cycle in women suffering from PMDD, there is an increase in serotonin uptake during the premenstrual period. Furthermore, increased serotonin transporter correlated with increased depressive symptoms. This indicates that the key may be the change in extracellular serotonin levels itself (52).

Another theory proposes the thesis that SSRIs promote an enzyme necessary for the production of allopregnanolone, and this enzyme is responsible for the immediate effect (53), which would explain the achievement of rapid clinical effects after brexanolone administration (54).

Within the allopregnanolone pathway, the enzyme 5α-reductase initiates the transformation of progesterone into 5α-dihydroprogesterone (5α-DHP). Subsequently, another enzyme, 3α-hydroxysteroid dehydrogenase (3α-HSD), facilitates the conversion of 5α-DHP to allopregnanolone (55, 56).

Progesterone can also be transformed into 5β-DHP with the enzyme 5β-reductase. Subsequently, 3α-HSD acts on 5β-DHP to produce pregnanolone (57).

Allopregnanolone and pregnanolone are positive allosteric modulators of GABAA, enhancing its function. Conversely, their isomers, isoallopregnanolone and epipregnanolone, are negative allosteric modulators, thereby inhibiting GABAergic neurotransmission. Dehydroepiandrosterone (DHEA) is another pregnanolone derivative and negative allosteric modulator, which potentially may compete with allopregnanolone for the substrate. Furthermore, a potential mechanism for PMS/PMDD could involve higher levels of negative allosteric modulators compared to positive allosteric modulators (57, 58).

Griffin et al. suggest that SSRIs (fluoxetine, paroxetine and sertraline were included in the study) may modulate the activity of neurosteroidogenic enzymes by enhancing their substrate affinity. For instance, they propose that SSRIs could increase the affinity of 3α-HSD for 5α-DHP, potentially augmenting its function. The specific mechanism of SSRIs influence on enzyme is currently unknown (51).

The mechanism of action of SSRIs in managing PMS/PMDD is convoluted, encompassing the modulation of GABA via neuroactive steroids. The SSRI’s impact on neuroactive steroid levels involves processes such as the redirection of biosynthetic pathways from progesterone towards neuroactive metabolites. Additionally, substrates are directed towards enhancing GABAA function positively, while competitive inhibition of enzyme substrates also plays a role. These mechanisms may contribute to the modulation of neuroactive steroid levels, suggesting the impact of SSRIs in addressing PMS/PMDD symptoms (58, 59).

### 2.3 Inflammation in PMS

One clue to the development of the disorder is immune dysregulation in women experiencing PMS. A study by Gold et al. revealed elevated levels of hs-CRP in women with PMS, indicating an immune component to the disorder (60). This study confirms the theory about the role of inflammation in its development, but there is insufficient evidence indicating a central effect of these substances in PMS.

The strong correlation of hsCRP with abdominal pain may suggest a local inflammatory process. Still, the central levels of cytokines are unknown, even though hsCRP was associated with mood disorders in the study. Unfortunately, the study had several limitations that could impact the CRP result: the patients’ status, prevalence, and BMI at the time the samples were taken were not considered (61). A relevant study by Puder et al. demonstrated that regardless of BMI, hsCRP levels are similar in women with high BMI and those within normal limits, and the course of low-grade inflammation is independent of BMI (62). It’s important to note that the study sample included only 15 women. Furthermore, hsCRP concentrations correlated once again with women’s mood, and hsCRP level itself was highest during the early follicular phase, associated with physiological processes. Another study, which excluded conditions such as smoking and a history of mood disorders, provided more robust evidence by demonstrating elevated levels of inflammatory cytokines in affected women (including IFN-gamma, IL-2, IL-10, IL-12, IL-4) (63). However, the study did not clarify the important time criterion for the appearance of these markers in the blood. The markers themselves, such as IFN-gamma, indirectly indicate T-lymphocyte activity, with correspondingly elevated IL-1 levels, highlighting the interconnectedness of anti- and pro-inflammatory factors. An additional argument supporting the importance of inflammation is the results of treatment of selected PMS using anti-inflammatory drugs (64).

### 2.4 HPA axis in PMS

Meta-analysis by Klusmann et al., showed that the HPA axis exhibits stronger reactivity during the luteal phase compared to the follicular phase (65). This is also linked to elevated cortisol levels during the luteal phase. Additionally, Hou et al. found that there is a blunted morning cortisol response in PMS (66). The dysregulation of the HPA axis may be caused by cyclical stressors experienced over time. In addition, PMS has been found to result in an impaired cortisol response to stress (67). Affective disorders are also linked to altered HPA axis function (68). It is important to note that the cortisol response and sympathetic nervous system response are impaired in PMS, but only during the luteal phase (69). However, the study by Schmidt et al. mentioned earlier does not provide enough evidence to determine whether it is progesterone alone via allopregnanolone, or both progesterone and estrogen, that contribute to this dysregulation. However, the available data suggest that estradiol-containing drugs may be effective in improving HPA function, as demonstrated by the improvement in function following estrogen administration (70). In addition, regulation of progesterone levels may prevent abnormal adaptations of GABA-A receptors and thus prevent changes in the HPA axis.

### 2.5 Prolactin in PMS

In the context of the etiology of PMS, the role of prolactin was also considered. Studies with bromocriptine provide indirect evidence for the effect of prolactin on PMS (71, 72). Additionally, higher prolactin concentrations are observed during the luteal phase, which is associated with PMS symptoms (73). Elevated levels of prolactin have been linked to mastalgia, and decreasing these levels has been shown to result in clinical improvement in patients (74). Based on the concentrations of estrogen and progesterone, high levels of prolactin may exacerbate PMS symptoms, in line with the theory proposed by Carroll and Steiner (72).

### 2.6 Genes in PMS

Genetic studies have not provided clear conclusions regarding the specific genes that are reliably involved in the development of PMS. However, family studies suggest a discernible genetic component and align with the theory of the existence of a subgroup of susceptible patients (75). Research conducted by Widholm et Kantero found that children of mothers with PMS have a higher likelihood of developing the disorder (76). Additionally, a study on monozygotic and dizygotic twins highlighted a greater than 40% probability of developing the disorder if one of the twins suffers from PMS (77).

**Figure 3:**
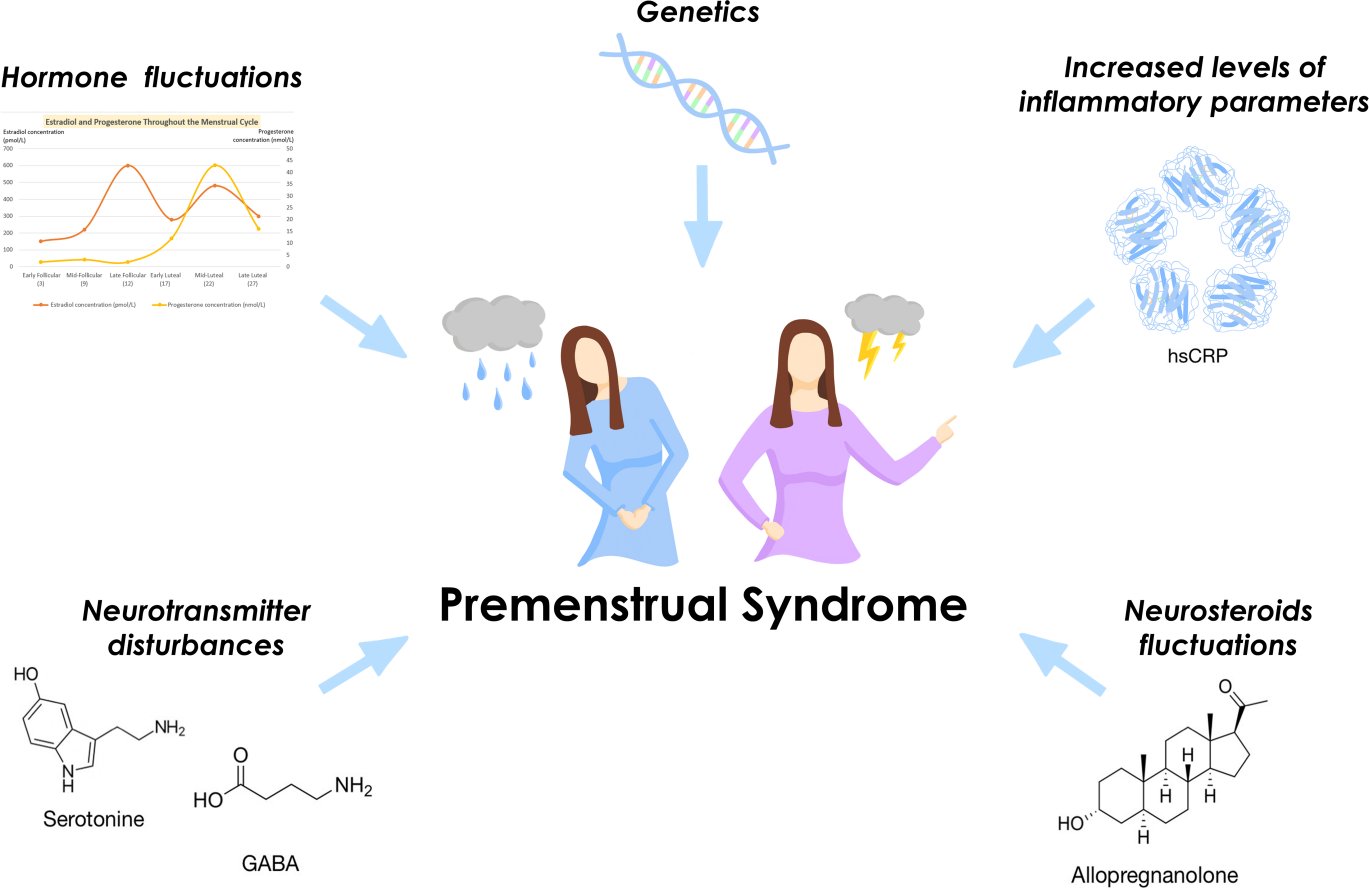
Selected factors influencing the development of PMS.

Although etiopathogenesis remains incompletely understood, studies on PMS markers and advancements in imaging techniques provide a rationale for the use of many drugs in the treatment of PMS (**Figure 3**).

## 3 Hormone treatment

Hormone treatment aims to eliminate fluctuations in sex hormones during the menstrual cycle. This can prevent adaptive changes in the Central Nervous System that occur under the influence of progesterone and estrogen derivatives. Theoretically, this could eliminate a group of women particularly sensitive to hormonal fluctuations. The attempted maladaptation of GABA-A receptors to allopregnanolone could be prevented by the absence of a progesterone peak (34, 78). Eliminating this phenomenon could potentially increase serotonin levels in women who suffer from PMS (50). Based on these interactions, monophasic COC preparations seem to be a better treatment option than multiphasic preparations. Biphasic and triphasic formulations gradually increase the amount of gestagens in the second half of the cycle, corresponding to the physiological fluctuations of sex hormones. This removes the progesterone peak, making abnormal adaptation of the body impossible. Monophasic preparations are recommended for controlling mood disorders during PMS, according to guidelines (10). A more detailed description of endocrine disruption is described above.

### 3.1 Contraceptive treatment

The most effective drug in the oral contraceptive (OC) group seems to be formulations containing ethinylestradiol and drospirenone. This preparation is FDA-approved for the treatment of PMDD (79). These drugs are intended to improve the patient’s condition through several mechanisms, including the suppression of ovulation, which results from the stabilization of hormone levels by both components of the pill. Theoretically, this is also expected to lead to an improvement in mood. The preparation is also intended to have an anti-androgenic effect, which would reduce symptoms such as irritability and aggression. However, the role of androgen hormones in PMS is not yet fully understood. Eriksson et al. found higher serum testosterone concentrations in women with premenstrual symptoms regardless of cycle (80), while another study (81) found no differences in testosterone concentrations between sick and healthy women. However, it should be noted that the latter study was limited by a small sample size. In addition, the heightened levels of DHEA during the periovulatory period in women with PMS highlight the significance of neurosteroids in the disorder. DHEA is a precursor for the synthesis of neurosteroids and has a protective effect on the CNS (82). However, the concentration of DHEA is higher for a short period during the cycle, indicating a different DHEA processing pathway in affected women.

Additionally, drospirenone is responsible for the anti-androgenic effect in the cited preparation (83). This substance is a progesterone derivative with up to 10 times the anti-androgenic effect. Drospirenone has been found to have a beneficial effect in reducing PMS and PMDD symptoms due to its antagonism to the mineralocorticoid receptor (84). This substance is an analog of spironolactone, a diuretic, which has been shown to nullify symptoms related to water retention and also has mood-enhancing effects (85). It is important to note that in the natural cycle, progesterone competes with aldosterone for access to the mineralocorticoid receptor, thereby antagonizing its action. While most progesterone analogs do not mimic this action, drospirenone is an exception. Spironolactone inhibits the action that results from the earlier dominance of estrogens in the cycle, which leads to the promotion of angiotensin formation (86). This is particularly relevant because angiotensin is responsible for various changes in the body, including its influence on the Central Nervous System. For instance, it regulates acetylcholinergic conductance (68). The earlier-mentioned improvement in mood after spironolactone may be correlated with its ability to lower and normalize progesterone concentrations (87). This suggests that spironolactone may block the body’s abnormal adaptation to progesterone and allopregnanolone. Drospirenone is an analog of spironolactone that performs the important function of progesterone in the periphery more effectively, with anti-androgenic and anti-mineral corticosteroid actions. Additionally, drospirenone lowers the concentration of progesterone in the body, which prevents abnormal adaptation reactions in the CNS. The initial studies on drospirenone were inconclusive. The relatively long period of placebo intake (21/7 days) may have been related to these observations: improvements in aspects of acne, appetite and hunger, and breast pain, but no significant improvement in mood was achieved (88, 89). Only studies using a shorter duration of placebo intake (24/4 days) demonstrated significant improvement in physical symptoms such as breast tenderness, swelling, bloating, headaches, and muscle pain, as well as mood. However, the authors highlighted that previous studies on the use of contraception in PMDD indicate its superiority in treating physical symptoms over mood, where SSRIs are still more potent (79). Lopez et al. (2008) demonstrated significant improvements in productivity and social relationships following a three-month treatment with ethyl estradiol and drospirenone (90). Additionally, this drug can reduce the risk of PMDD recurrence (79). To achieve maximum treatment efficacy, it is recommended to administer the specified preparation for 24 days with a 4-day interval. The preparation contains 20ug of ethinylestradiol and 3mg of drospirenone. If treatment is ineffective, increase the dose of ethinylestradiol to 30ug and take the preparation in a cycle of 21 days with a 7-day interval, along with 3mg of drospirenone. According to the cited data, a shorter medication interval improves the mood of sufferers (10, 79). Therapy with this contraceptive, like other drugs, may cause side effects (**Table 1**). Patients may experience nausea, breast pain, and intermenstrual bleeding (95).

**Table 1:** Collected research on hormone treatment in PMS.

Author, year of publication	Substance, dosage form and total daily dose	Study design	Dosage, total duration of administration	First test group	Second test group	Third test group	
Pearlstein et al. 2005 (79)	oral contraceptive (OC) containing drospirenone 3 mg and ethinyl estradiol (EE) 20 µg pills	multicenter, double-blind, placebo- controlled, crossover	3 mg drospirenone + 20 EE µg/dayfrom day 1 of the cycle to day 24 during 3 menstrual cycles	**OC formulation (3 cycles)->washout (1 cycle) -> placebo (3 cycles)**34 women [18-40 years old](14 completed study)	**placebo (3 cycles) -> washout (1 cycle) -> OC formulation (3 cycles)**30 women [18-40 years old](11 completed study)	**-**
Freeman et al. 2001 (88)	oral contraceptive (OC) drospirenone (DRSP, 3 mg) and ethinyl estradiol (EE, 30 µg)	multicenter, double-blind, randomized, placebo- controlled	3 mg DRSP + 30 µg EEduring 3 cycles	**DRSP/ EE**42 women [18-40 years old]	**Placebo**40 women [18-40 years old]	–
Graham and Sherwin 1992 (89)	triphasic OCethinyl estradiol 0.035 mg, norethindrone 2 mg tablets	double-blind, controlled	ethinyl estradiol 0.035 mg from days l-21 and norethindrone,0.5 mg during days l-7, 1 mg during days 8-16,and 0.5 mg during days 17-21. 1 tablets of O.C. /dayduring 3 cycles	**OC**20 women [18-35 years old]	**Placebo**25 women [18-35 years old]	**-**
Lundin et al. 2016 (93)	combined oral contraceptive (COC)1.5 mg estradiol and 2.5 mg nomegestrolacetate pills	investigator- initiated, multi-center, randomized,double-blinded, placebo-controlled	COC pill/ dayfrom day 1 to day 24 of cycle during 3 cycles	**COC**84 women [18 – 35 years old]	**Placebo**94 women [18 – 35 years old]	**-**
Halbreich et al. 2011 (105)	oral contraceptive (OC) Levonorgestrel 90 mcg/ethinyl estradiol 20 mcg (LNG/EE) tablets	multicenter, randomized, double-blind, placebo- controlled	4 consecutive 28-day pill packsfor 112 days	**LNG/EE**186 women[18-49 years old](127 women completed)	**Placebo**181 women[18-49 years old](128 women completed)	–
Comasco et al. 2021 (117)	Ulipristal acetate (UPA) 5 mg	investigator- initiated, multicenter, double-blind, randomized, parallel-group	5 mg UPA/ day for 28 daysduring 3 menstrual cycles	**UPA**48 women [18-46 years old](41 women icluded in analys)	**Placebo**47 women [18-46 years old](39 women icluded in analys)	**-**
Bäckström et al. 2021 (142)	Sepranolone 10 or 16 mg	parallel, randomized, double-blind, placebo-control	10 or 16 mg sepranolone/ dayduring 14 days prior to the next estimated menstruation for 3 menstrual cycles	**Sepranolone 10 mg**64 women [18-45 years old](50 women icluded inanalys)	**Sepranolone 16 mg**68 women [18-45 years old](49 women icluded inanalys)	**Placebo**70 women[18-45 years old](44 women icluded in analys)
Author, year of publication	Method of data collection	Outcomes	Adverse events/ side effects	Limitations	Overall effect	
Pearlstein et al. 2005 (79)	Daily Record of Severity of Problems (DRSP) scores	Significantly reduce PMS symptoms and improved DRSP total score while using drospirenone/EE than placebo. the greatest improvementswere found for the physical symptoms of breast tenderness,swelling, bloating, headache and muscle pain and also improved mood	7 womens discontinued the study due to AEs (4 while using drospirenone/EE and 3 while taking placebo)Reasons for discontinuation from drospirenone/EE were spotting/dysmenorrhea/vomiting, breast tenderness/clumsiness/nervousness, spotting and severe nervousness/increased irritability,and from placebo were severe nervousness/increased irritability, migraine	-the sample size was modest,-the length of the study led to considerable subject attrition-OCs potentially change the characteristics of the menstrual cycle (e.g., cycle length, increased intermenstrual bleeding), which could potentially unblind both subjects and study personnel	positive	
Freeman et al. 2001 (88)	Calendar of Premenstrual Experiences (COPE) scale Beck Depression Inventory (BDI) and the Profile of Mood States (POMS)	There was greater improvement in the total COPE scores in the DRSP/EE group compared with the placebo group (appetite, acne, and food cravings) but the difference did not reach statistical significance	The most commonly reported adverse events were nausea, headache, sinusitis, upper respiratory infection, malaise, and depression in both groups	-high placebo response rate-the COPE instrument, which was used as a primary outcome measure, has a range of 0–3, which may not have been sensitive enough to detect differences between active and placebo treatment over time	positive	
Graham and Sherwin 1992 (89)	the Daily Ratings Form (DRF) visual analogue scales (VAS)	Premenstrual breast pain and bloating were significantly reduced with O.C. there were no beneficial effects of theO.C. over placebo for any of the mood symptoms.	decreased sexual interest after starting treatment with O.C.	-small sample size,-women were only observed for 3 menstrual cycles	positive	
Lundin et al. 2016 (93)	the Daily Record of Severity of Problems (DRSP) the self-rated version of the Montgomery- Åsberg Depression RatingScale (MADRS-S)	COC use is associated with small but statistically significant mood side effects in the intermenstrual phase.Significant treatment by menstrual cycle phase interactions were noted for mood swing, irritability and anxiety, also minimal for mood change, depression, and sense of being overwhelmed	COC users reported a clinically significant mood worsening and 15 placebo users	the placebo response was substantial, and many women also improved throughout the trial.	slightly positive	
Halbreich et al. 2011 (105)	Daily Record of Severity of Problems (DRSP)Work Limitations Questionnaire (WLQ)	LNG/EE may be useful for managing the physical, psychological and behavioral symptoms and loss of work productivity related to PMDD.LNG/EE decreased PMDD symptoms in the late luteal phase as well as on worst symptomatic days, reduced severity of the predefined physical, depressive and irritability symptom clusters and reduced work limitation.	Headache was the most commonly reported AE with both treatments. metrorrhagia, menorrhagia and vaginal and uterine hemorrhage and flulike syndrome more often in LNG/EE group	-a high number of subjects failed screening (maybe because of challenging to complete a daily-rating questionnaire for 7 months)-high placebo response(the placebo run-in cycle only partially addressed this issue),-small sample size	positive	
Comasco et al. 2021 (117)	Daily Record of Severity of Problems (DRSP)EuroQoL visual analogue scale (EQ- VAS)Montgomery-Åsberg Depression Rating Scale, self-rated version (MADRS-S)	Treatment effects were also noted for the DRSP depressive symptom subscale (anger/irritability), but not for the DRSP physical symptom subscale.UPA could be a useful treatment for PMDD, particularly for the psychological symptoms of PMDD	7 women in the UPA group discontinued because of mild/ moderate side effects (headache, fatigue, and nausea) and 3 women in the placebo group discontinued because of depressive symptoms or anxiety.Nausea was significantly more common among women in the UPA group than in the placebo group.Other most commonly reported side effects in the UPA group were headache, nausea, and fatigue.	-small sample size,-women were only observed for 3 menstrual cycles	positive	
Bäckström et al. 2021 (142)	Daily Record of Severity of Problems (DRSP) scale in an eDiary	Sepranolone 10 mg reduced PMDD symptoms significantly more than placebo. The effect of sepranolone 16 mg dosage did not statistically differ from placebo.10 mg sepranolone could ameliorate negative mood symptoms, improve distress and impairment occurring in the premenstrual phase to a greater degreethan placebo	14 subjects discontinued the study due to a treatment-emergent adverse event (3 in placebo group, 5 in sepranolone 10 mg group and 6 in sepranolone 16 mg group)The most prevalent AE was injection site pain (8 in sepranolone 16 mg group).	-small sample size-women were only observed for 3 menstrual cycles	positive	

The use of oral contraceptives that contain only progesterone is not recommended for the treatment of PMS and PMDD symptoms. This is because such therapy may exacerbate mood fluctuations and other PMS-related symptoms (91, 96). Evidence supporting this position comes from a study that found that patients with mood disorders have higher levels of progesterone in their blood compared to the control group (97). It is known that the development of progesterone-induced mood disorders is strongly dependent on the individual’s sensitivity to the hormone, its concentration in the blood, and the timing of exposure. It is worth noting that it is progesterone administered in doses that mimic the luteal phase, and therefore in lower concentrations comparatively to pregnancy, that may be associated with mood side effects in OC users (91, 98–100). In contrast, during pregnancy, high concentrations of the substance exhibit anti-anxiety and sedative effects (101–104). Consequently, the effectiveness of progesterone in alleviating premenstrual symptoms strongly depends on its blood concentration. In conclusion, the use of progesterone alone in the treatment of PMS and PMDD does not show the same efficacy as therapy with oral contraceptives containing drospirenone with ethinylestradiol. Furthermore, it appears that premenstrual symptoms may be induced by the use of progestogen as part of hormone replacement therapy (105). In terms of targeted treatment, dutasteride may be a more suitable option as it inhibits the conversion of progesterone to allopregnanolone (34). However, there is limited data on the efficacy of this substance, and as an androgen, it may have negative effects on male fetal development in women who are planning pregnancies.

The use of oral contraceptives containing only estrogens is not recommended for the treatment of PMS and PMDD symptoms. Studies suggest that such preparations may be ineffective in alleviating premenstrual symptoms or may even worsen them (106). In addition, it has been shown that estrogens are significantly associated with an increased risk of endometrial cancer. However, this risk can be effectively reduced by concomitant use of progesterone (106). Therefore, a more effective and safer approach would be the use of combination preparations, such as the OC and COC preparations cited earlier (107).

Researchers have also considered the issue of the placebo-drug interval. Although OC treatment is effective, it does not fully eliminate hormonal fluctuations. This may be related to a treatment regimen involving a placebo (92). The use of COC - continued contraception - could eliminate LH, FSH, oestradiol, and progesterone fluctuations, thus improving patient comfort (108). Halbreich et al. (2011) studied the effectiveness of levonorgestrel (LNG) 90 mcg/EE 20 mcg for 4 cycles of 28 days. The study found that over half of the patients experienced a significant improvement, defined as a 50% reduction in symptom intensity (92). Furthermore, as the therapy duration increased, more patients responded positively to the treatment. In the initial cycle, during the late luteal phase, typically associated with the onset of symptoms, there was a decrease in symptom intensity according to the DRSP scale. However, according to Freeman et al.’s analysis of studies, the efficacy of COC treatment is similar to that of SSRIs. It should be noted that the effect of COC treatment is not as well demonstrated for low symptom severity (109). One possible reason for the PMS trials showing less clear outcomes than the PMDD trials is that the PMS trials had lower criteria for symptom severity at the start of the study. This could have made it harder to see the differences in how much the LNG/EE and placebo groups improved, compared to the PMDD trials where the participants had more severe symptoms and more room for improvement. It is important to note that these studies are limited by high responses in the placebo group, ranging from 27-53% for PMDD. Additionally, COC treatment offers better control of bleeding days and reduces pain associated with the 5 most severe days of the cycle (110). Therefore, these drugs appear to be particularly effective in more severe cases of PMS - PMDD, especially when physical symptoms are inadequately controlled. COCs have been shown to improve patients’ mood and physical symptoms.

It is noteworthy that the use of a levonorgestrel-releasing IUD may increase stress sensitivity. Women using this type of IUD exhibited significantly higher blood cortisol levels than those who took oral levonorgestrel in combination with estrogen. This phenomenon may be due to the potential effect of this type of contraception on increasing autonomic system reactivity to stimuli such as stress (111). It is worth noting that several studies have suggested that the use of levonorgestrel-releasing IUDs may worsen mood disorders (111–115). In conclusion, it is important to note that the effectiveness of levonorgestrel in alleviating PMS symptoms appears to depend on its method of administration. Oral formulations containing levonorgestrel demonstrate greater efficacy than IUDs, which may even exacerbate symptoms associated with the disorder.

### 3.2 GnRH agonist treatment

Alongside oral contraceptives, gonadotropin-releasing hormone (GnRH) agonists also play a significant role in the treatment of PMS and PMDD. The mechanism of action involves inhibiting the central hypothalamic-pituitary-ovarian system, which leads to the inhibition of ovulation. This has been confirmed in studies (116). Inhibiting ovulation is expected to reduce hormonal fluctuations in the menstrual cycle. However, it is important to note that these drugs induce a menopausal state, which can cause symptoms such as bone mass loss and hot flashes. To minimize the side effects of therapy, progestogens or tibolone are often added. Another option is to use a progesterone receptor blocker, which, if given early enough in the cycle, also prevents ovulation. Ulipristal acetate is a progesterone receptor blocker used to treat uterine myoma. Receptors for progesterone are present in the hippocampus and frontal cortex (117). This highlights the significance of this steroid in the disorder. Blocking its receptor would prevent interactions between progesterone and the genome, inhibiting potential negative changes (118). Comasco et al. (2020) conducted a study that found that taking ulipristal significantly improved psychiatric/mental symptoms in PMDD sufferers compared to placebo. The study lasted for three months and outcomes were measured using the Daily Record of Severity of Problems (DRSP) scale (93).

The group of GnRH agonists match the efficacy of first-line drugs - SSRIs. Due to their induction of the perimenopausal state/suppression of estrogen and progesterone synthesis (mentioned above), they cause several side effects characteristic of the menopausal period (119) These side effects limit the duration of therapy to a maximum of 6 months, with the main limitation being the loss of bone mass (120). To address the problems of therapy with GnRH analogs, attempts are being made to use add-back hormone therapy to reduce the incidence of side effects (121).

However, this is a controversial approach due to the etiology of PMS in which hormonal fluctuations seem to be the predominant problem. In theory, this could lead to counteracting the therapeutic effect of GnRH. Progestogens themselves can trigger a worsening of mood in women, presumably through their effect on the GABAa receptor (82). According to Schmidt et al.’s theory, not only progesterone but also estradiol administered alone can induce a relapse of PMS symptoms (33). Similar conclusions were reached by Leathear et al. in whose study of GnRH with add-back hormone therapy as many as nine out of 20 subjects discontinued therapy, when in the case of the GnRH analog alone it was three out of 20, including only one for medical indications. The entire study lasted six months and showed that people on add-back hormone therapy did not achieve clinically significant improvements compared to placebo (121). Given that progestogen was given only for one week into a cycle in this study, it is debatable to use add-back therapy alone with estrogen. This would eliminate the effect of progestogen, which, when administered during the luteal phase, can mimic premenstrual symptoms (122). On top of this, a study by Erkkola et al. indicates that progestogen supply every 3 months for 14 days was sufficient in menopausal women to prevent endometrial hyperplasia (123). Furthermore, in a study by Mezrow et al, it was shown that add-back estrogen was also effective, however, each time Medroxyprogesterone acetate (MPA) was administered for 10 days every 4 cycles, this was accompanied by a worsening of mood (124). Further studies, on a group of PMS and PMDD patients, are needed to confirm these reports and effectively apply this type of therapy in selected patients. In agreement with the data presented here correlates with the study by Segebladh et al. who showed that the addition of HRT in women with PMDD specifically worsened the control of mood-related symptoms, however, the addition of 1.5mg of oestradiol alone (gel, daily) least interfered with the outcome of leuprolide acetate treatment (125). Furthermore, the higher the concentration of estradiol relative to progesterone in the other groups of the study, the more pronounced the premenstrual symptoms were. This evidence indirectly suggests that the co-occurrence of hormones in the cycle potentiates their interaction with mood, lowering it even more strongly in predisposed women. This also challenges the approach that progesterone metabolites alone are crucial for the development of the premenstrual disorders (126).

There are only a limited number of studies that have examined the effect of GnRH agonists along with add-back hormone treatment, which makes it difficult to draw clear conclusions. A meta-analysis conducted by Wyatt et al. indicated that add-back therapy does not reduce the effectiveness of GnRH agonists based on several studies (127). However, more recent studies have raised doubts about these findings. Ultimately, a high placebo effect, typical of PMS studies, reduces the quality of the results. The mere administration of a placebo may suggest the reappearance of hormone fluctuations and subsequent symptoms in female patients (128). Additionally, it should be noted that the effectiveness of GnRH therapy decreases in patients with a co-existing psychiatric diagnosis, which is more frequent in the PMS population than in the general population. There is no doubt that the problem of adverse effects of GnRH analogs requires replacement therapy, and studies suggest that the best combination would be oestradiol alone with progesterone administered approximately every 3–4 cycles. However, it should be noted that progesterone administration may be accompanied by an increase in symptoms, and this should be brought to the patient’s attention when attempting such therapy. On top of this, the small amount of evidence limits such an approach.

When discussing GnRH agonists, GnRH antagonists should also be considered. GnRH antagonists rapidly inhibit pituitary gonadotropin secretion through competition for GnRH receptors, eliminating the initial stimulatory phase typical of agonists. They have indications, among others, in the treatment of endometriosis (129).

The reason why they can be considered for use is their rapid onset of action and rapid return of pituitary function after cessation of therapy (130). GnRH agonists must be administered for a longer period and on a relatively continuous basis to maintain their effect. While the therapeutic regimen would not differ in terms of continuity of therapy in the case of GnRH antagonists for PMS and PMDD, these drugs are more predictable in their use. However, they can be expensive and may require hormone replacement therapy (131). While there are no studies that discuss the use of these drugs for PMS and PMDD, they may become more convenient for clinicians to use in the future.

### 3.3 Future directions

As blocking the synthesis of progesterone metabolites, including allopregnanolone, has been found to provide relief to patients with PMDD, it is important to attempt to normalize the concentration of this substance (132). Low concentrations of allopregnanolone can worsen mood in certain cases (133). Conversely, when its concentration peaks, its activity has been associated with a decrease in amygdala impulsivity (134).

The appearance of allopregnanolone appears to affect GABA receptor modulation, with only high concentrations being beneficial in a therapeutic context. This is observed in the group where a paradoxical anxiety mechanism is described at low concentrations (135). However, a potential issue in this scenario is determining the appropriate timing for terminating treatment with the drug. The decrease in allopregnanolone concentration appears to trigger the re-conformation of the GABA receptor (39). Our current knowledge is insufficient to use the substance that is blamed for mood fluctuations. The abnormal receptor response appears to underlie the pathogenesis of the disorder, given the somewhat common paradoxical mechanism of action of GABA-A modulators, which also involves the action of benzodiazepines (136) and ethanol (135). Another issue is blocking progesterone metabolism to inhibit allopregnanolone synthesis. SSRI drugs appear to normalize allopregnanolone concentrations, which may explain their rapid effect in women with premenstrual symptoms (137). In contrast, isoallopregnanolone is a negative modulator of GABA, unlike allopregnanolone, but its effect on GABA is small (138, 139). In studies conducted on rats, isoallopregnanolone was found to reverse the effects of allopregnanolone (140). Additionally, even a half dose of isoallopregnanolone was able to reverse the sedative effects of allopregnanolone as well as the SEV test, which measures the intensity of anesthesia (141). To ensure the drug’s effectiveness, it should be administered based on the predicted concentrations of allopregnanolone during the cycle. This means that its concentration should be highest in the later luteal phase to counteract the fall of allopregnanolone. Bäckström et al.’s study showed the greatest improvement in patients with the highest concentrations of the drug in the late luteal phase. Despite methodological errors, such as administering the drug outside of the late luteal phase, it still demonstrated efficacy (142). A recent study found that women who took isoallopregnanolone had a lower incidence of PMDD symptoms than those who took a placebo (94). However, both studies have methodological problems. In the first study, inaccurate adjustment of the drug’s administration timing to the luteal phase and an unselective inclusion criterion were noted. Women with symptoms outside the luteal phase were also admitted. Furthermore, the initial analysis in the second study only considered the 5 days of the cycle with the most severe symptoms. It was not until the extension to 9 days that a significant benefit from the drug was observed. Additional research is required to establish definitive conclusions. Currently, it is understood that isoallopregnanolone is particularly effective in improving mood, reducing tension, and alleviating anxiety. The medication appears to be beneficial for patients with mental disorders during their menstrual cycle.

### 3.4 Conclusion

In conclusion, for PMS therapy, oral contraceptives containing drospirenone and ethinylestradiol (at a dose of 3 mg drospirenone and 20 mcg ethinylestradiol) are the most effective. If bleeding and abdominal pain are not controlled, an alternative solution is to use COCs with levonorgestrel and EE. Transdermal patches may be used as an alternative to oral contraceptive pills for patients who have difficulty taking them regularly. However, the effectiveness of transdermal patches is still a matter of debate (106). It is not recommended to use formulations that contain only progesterone or only estrogen. The available research on the effectiveness of new treatments that selectively target progesterone and its metabolites is insufficient to draw firm conclusions.

It is important to note that many PMS and PMDD symptoms, including breast tenderness, depression, and headaches, can occur as side effects of taking contraception, which limits the effectiveness of this approach (143). Although side effects were rare in most of the studies cited, it is still necessary to consider alternative therapeutic approaches for premenstrual disorders. It is worth noting that the preparations discussed above mainly affected physical symptoms, and only some had a clear effect on improving mood.

## 4 Antidepressant medication

The treatment of PMS and PMDD uses drugs from the SSRI group, which block serotonin reuptake in the presynaptic area. This leads to an increase in serotonin concentration in the synaptic cleft, increasing serotonergic neurotransmission (46).

According to the latest guidelines from the Royal College of Obstetricians and Gynaecologists, SSRIs should be used as first-line drugs in the pharmacotherapy of severe PMS (144). Primarily because they are considered most effective in alleviating the anxiety and irritability symptoms characteristic of the disorder (145). Studies on the use of SSRIs to treat PMDD have shown a beneficial effect of the therapy in 60% to 90% of patients, with a range of 30% to 40% to placebo (22).

The exact percentages depended on the criteria the patients met. Among the most important were the severity, type, and number of symptoms reported (50). Such criteria limit the determination of the percentage beneficial therapeutic effect for the entire group of women suffering from PMS.

An advantage of the use of SSRIs in the course of PMS, as opposed to their use in the treatment of depression, is their rapid effect, achieved even within days of starting medication. This indicates a different mechanism of activity than that observed in depression therapy, where measurable improvement can be observed after a few weeks of taking the drugs (46, 146, 147).

The rapid effects of SSRIs in women with PMS or PMDD are likely due to their simultaneous effects on serotonin receptors and allopregnanolone levels in the brain, thereby indirectly modulating GABAA receptor function. Increasing the efficiency of DHP conversion to allopregnanolone SSRI group drugs also alters the levels of this neurosteroid (22, 148).

The swift effects of SSRIs in treating PMS and PMDD allow them to be used not only continuously, but also intermittently (only during the luteal phase) (149). Currently, there are no studies that show a clear difference in the efficacy of SSRIs in relieving PMS, comparing administration either continuously or only during the luteal phase. However, it should be noted that at this point the number of studies is insufficient to draw confident conclusions (46). Taking SSRIs only in the luteal phase avoids the withdrawal syndrome associated with long-term antidepressant use (150).

The choice of route of administration, i.e.: continuously or only in the luteal phase in women with severe PMS or PMDD without comorbidities, may be based on patient or physician preference and individual experience of side effects occurring in a given patient (151). It is worth mentioning that it is necessary to gradually discontinue the intake of SSRI drugs when they are administered continuously (144). Otherwise, there is a risk of adverse effects, the most common of which are nausea and weakness. Marjoribanks et al. showed that there is a correlation between the dose of an SSRI and the appearance of side effects. It seems that higher doses of the drug are associated with an increased likelihood of experiencing its side effects (46).

The entire group of SSRI drugs can be used to treat premenstrual symptoms. According to the Marjoribanks et al. (46), too few studies have been conducted using a specific drug from the SSRI group to indicate significant differences in the effectiveness of PMS treatment. The choice of drug should be based on the individual clinical situation of the patient, this is to minimize the severity and frequency of adverse effects.

In the literature, it is possible to distinguish SSRI drugs for the treatment of PMS such as fluoxetine, sertraline, paroxetine, citalopram, and escitalopram, the first 3 of which are approved by the FDA (50).

The criteria for selecting patients for the study were most often similar. They included aspects such as an age range of 18 to 45 years, regular menstrual cycles of 22 to 35 days, and evidence of probable ovulation. They also included meeting the criteria for a diagnosis of PMS/PMDD, and the absence of psychiatric comorbidities. Side effects that can occur with specific SSRI drugs are typically common to the entire group. Such adverse effects as decreased libido, nausea, weakness, drowsiness, fatigue, and sweating can be mentioned (46, 50).

### 4.1 Fluoxetine

Preclinical studies suggest that low doses of fluoxetine may increase allopregnanolone concentrations in the brain (152).

A pilot study on the use of fluoxetine to treat PMS conducted on 40 women showed the potential to alleviate the emotional symptoms of PMS. The administration of a dose of 10 mg/day during the luteal phase of the menstrual cycle, 7 days before the probable date of menstruation, was found to yield the most favorable outcomes. This led to a reduction of emotional PMS symptoms by more than 40% in 70% of the study participants, in comparison to placebo. The study was a randomized, double-blind, placebo-controlled trial (152).

Another double-blind pilot study of 39 women reported the efficacy of fluoxetine compared to placebo and calcium. Fluoxetine and calcium carbonate were administered for a period of 4 menstrual cycles. The dose taken by the patients was 10 mg of fluoxetine twice daily. Calcium carbonate was administered at 600 mg twice a day. The study shows noticeable benefits in treating PMS with fluoxetine. Efficacy with calcium was significantly lower, although higher than with placebo. Limitations of this study were the significance achieved in only 2 of the 5 symptom assessment instruments and the small study sample. According to the authors, there is no need to further compare the efficacy of fluoxetine with calcium in the treatment of PMS (153).

A study by Hedayat et al. conducted on 100 women compared the efficacy of fluoxetine and buspirone in treating PMS. The study was single-blind. The doses the patients were given were 20 mg/d of fluoxetine in one group and 10 mg/d of buspirone in the other. In both cases, the administration period was 2 months. Both drugs showed significant efficacy in treating PMS, with no significant differences between them. The authors believe that buspirone may be a better choice for treatment, due to fewer side effects. However, a limitation related to the lack of a placebo group should be taken into account here (154).

Hunter et al. demonstrated that fluoxetine, used in the treatment of PMDD, had a faster and more effective impact on alleviating anxiety-related symptoms compared to CBT therapy. However, after six months, the effectiveness of CBT therapy and fluoxetine use yielded similar results. The combination of both treatments showed no additional benefits. Such findings may contribute to better-tailoring therapy to the unique requirements of each patient. The female participants in the study were administered a daily dose of 20 mg for six months, and the study included forty-five women (155).

A study comparing the efficacy of fluoxetine with placebo in the treatment of PMDD showed that of the side effects, only decreased libido was observed with a statistically significant higher frequency among patients taking fluoxetine. Efficacy in alleviating physical symptoms was observed only among those administered a daily dose of 20 mg of fluoxetine. The reduction in the severity of problems was estimated at 38% for the 20 mg/d group, administering the drug daily only during the luteal phase (156).

According to a study from 2003, the difference in efficacy between a dose of 20 mg/d and 60 mg/d of fluoxetine was not statistically significant. In both cases, compared to placebo, efficacy was higher. At the 60 mg/d dose, adverse effects were more common (157).

Another clinical trial also proves there are no statistical differences between the efficacy of a 20 mg/d dose and a 60 mg/d dose in treating the physical symptoms of PMDD. Statistically significant differences were observed when tolerance to fluoxetine developed, favoring the 20 mg/d dose. At the 60 mg/d dose, patients were significantly more likely to discontinue treatment due to side effects (158).

Another study involving 405 women also reported that a 60 mg/d dose of fluoxetine resulted in a higher incidence of side effects compared to a 20 mg/d dose (159).

Fluoxetine, due to its high efficacy and the relatively high number of studies compared to other SSRI drugs in the treatment of PMS/PMDD, appears to be an appropriate form of medication as a first-line drug. It is worth noting that using the lowest effective dose is advisable, considering that, in selected studies, doses as low as 10 mg effectively controlled symptoms and carried a lower risk of side effects (**Table 2**).

**Table 2:** Collected research on fluoxetine treatment in PMS.

Author, year of publication	Drug, dosage form and total daily dose	Study design	Dosage, total duration of administration	First test group	Second test group	Third test group	Fourth test group
Maranho et al. 2023 (152)	Fluoxetine capsules 2 mg or 5 mg or 10 mg	randomized, double-blind, placebo-controlled pilot	2 or 5 or 10 mg/day,7 days before the first day of cycle to the first day of following cycle	**Fluoxetine** (2 mg)10 women[18-40 years old]	**Fluoxetine** (5 mg)10 women[18-40 years old]	**Fluoxetine** (10 mg)10 women[18-40 years old]	**Placebo**10 women[18-40 years old]
Yonkers et al. 2013 (153)	Fluoxetine capsules 20 mg	randomized, double-blind, placebo-controlled, parallel	2 x 10 mg/dayduring 4 cycles	**Fluoxetine**13 women[25-45 years old]	**Calcium carbonate**13 women[25-45 years old]	**Placebo**13 women[25-45 years old]	–
Nazari et al. 2013 (154)	Fluoxetine 20 mg	randomized, single-blind	20 mg/dayfor 2 consecutive moths	**Fluoxetine**50 women[18-49 years old](38 women were included in the analysis)	**Buspirone**50 women[18-49 years old](37 women were included in the analysis)	–	–
Hunter et al. 2002 (155)	Fluoxetine tablets 20 mg	randomized, open label	20 mg/dayduring cycle for consecutive 6 moths	**CBT**24 women[20-45 years old](21 women were included in theanalysis)	**Fluoxetine**21 women[20-45 years old](19 women were included in theanalysis)	–	–
Cohen et al. 2002 (156)	Fluoxetine capsules 10 mgor 20 mg	randomized, multicenter, double-blind,placebo-controlled, parallel-group	10 or 20 mg/day, during the luteal phaseand 3 cycles (in a double- blind manner)	**Fluoxetine** (10 mg)88 women[18-45 years old](77 women were included in the analysis)	**Fluoxetine** (20 mg)86 women[18-45 years old](64 women were included in the analysis)	**Placebo**88 women[18-45 years old](75 women were included in the analysis)	–
Steiner et al. 2003 (157)	Fluoxetine20 mg or 60 mg	randomized, double-blind, placebo-controlled, parallel	20 or 60 mg/dayduring 6 cycles	**Fluoxetine** (20 mg)104 women[18-45 years old](94 included in analysis)	**Fluoxetine** (60 mg)108 women[18-45 years old](85 included in analysis)	**Placebo**108 women[18-45 years old](94 included in analysis)	–
Steiner et al. 2001 (158)	Fluoxetine20 mg or 60 mg	randomized, double-blind, placebo controlled, parallel	20 or 60 mg/dayduring 6 cycles	**Fluoxetine** (20 mg)104 women[18-45 years old](95 included in analysis)	**Fluoxetine** (60 mg)108 women[18-45 years old](85 included in analysis)	**Placebo**108 women[18-45 years old](94 included in analysis)	–
Steiner et al. 1995 (159)	Fluoxetine20 mg or 60 mg	randomized, double-blind, placebo-controlled,(two-phase study design consisted of a single-blindwashout period)	20 or 60 mg/dayduring 6 cycles	**Fluoxetine** (20 mg)102 women[18-45 years old](96 included in analysis)	**Fluoxetine** (60 mg)106 women[18-45 years old](86 included in analysis)	**Placebo**105 women[18-45 years old](95 included in analysis)	–
Author, year of publication	Method of data collection	Outcomes		Adverse events/ side effects		Limitations	Overall effect
Maranho et al. 2023 (152)	Daily Record of Severity Problems Scale (DRSP)	All fluoxetine groups showed greater improvement.Scores decreased in all treatment groups, but most significantly in the Fluoxetine (10 mg) group.Participants in the 10mg/day group reported a reduction in symptoms exceeding 40%.		-headache (4 women in fluoxetine groups and 2 in placebo),-increased sleep duration (1 woman in fluoxetine group and 1 in placebo),-somnolence/sedation (2 women in fluoxetine group)-nauseas and decreased sleep duration (1 woman in fluoxetine group for each AE)		-small sample size-the order of natural cycle versus intervention cycle was not counterbalanced-markers of the GABAergic system or progesterone and its metabolites in plasma not included-diagnosis of emotional PMS over single cycle not two-short duration of the study	positive
Yonkers et al. 2013 (153)	Daily Record of Severity Problems Scale (DRSP), The Inventory of Depressive, Symptomatology, Premenstrual Tension Scale,Clinical Global Impression- Severity and Improvement scales,last-observation-carried forward method	In IDS-LOCF and PMTS-LOCF women in the fluoxetine group showed improvement in symptoms compared to the placebo group.		-nausea (1 woman in fluoxetine group, 4 in calcium group and 2 in placebo),-headache (1 woman in fluoxetine group, 3 in calcium group and 2 in placebo),-dry mouth (2 woman in fluoxetine group),-feeling spacy (2 woman in calcium group),-dizziness (1 woman in fluoxetine group and 2 in calcium),-fatigue (1 woman in calcium group),-irritability (1 woman in placebo group) and more*		-fluoxetine group had greater baseline severity on the DRSP-small sample size-recruitment via media advertisements-short duration of the study-single dosing regimen	positive
Nazari et al. 2013 (154)	The PMS diary,a self-assessment symptom rating scale	Both fluoxetine and buspirone demonstrated significant effectiveness in treating patients.		not investigated		-no placebo group-short duration of the study-single dosing regimen	positive
Hunter et al. 2002 (155)	The HADS,Causal attributions, The Coping Checklist, Ten-point Likert scales,Open-ended questions regarding the helpful and unhelpful aspects of the treatment,COPE measure	While both fluoxetine and the other treatment achieved similar overall effectiveness after 6 months, fluoxetine appeared to have a stronger effect on reducing anxiety symptoms.		gastrointestinal symptoms and loss of libido in fluoxetine group,more information not mentioned		-no placebo group-small sample size-recruitment via advertisements in newspapers and magazines-single dosing regimen	positive

### 4.2 Sertraline

A 1997 study observed significant improvement in PMDD symptom relief with sertraline administered continuously. The overall evaluation showed a great or very great improvement in 62% of those given sertraline and 34% of those in the placebo group (160).

A study by Freeman et al. on the use of sertraline to treat PMS found improvements in mood and relief of physical symptoms in women using sertraline. Doses ranged from 50 mg/d to 100 mg/d. Improvements occurred as early as the first month of treatment. The study was randomized, double-blind, and placebo-controlled (151).

In a 3-month, placebo-controlled comparison of sertraline and desipramine, the study revealed a significant advantage of the SSRI drug over the noradrenergic affinity drug. The degree of improvement was measured using the Penn Daily Symptom Report (DSR), indicating that symptoms decreased by more than 50% in 65% of the women studied (161).

According to a 2015 study, which investigated the efficacy of sertraline, including a placebo, on 188 women, treatment with this SSRI drug is not universally effective when administered *ad hoc*. The study utilized doses of 50 mg/d and 100 mg/d of sertraline (162).

A 2006 randomized clinical trial involving 314 women suggests the effectiveness of sertraline in alleviating moderate to severe PMS symptoms. Patients received sertraline throughout the luteal phase for the first two cycles, followed by continuous administration for one cycle and initiation of treatment at the onset of symptoms for one cycle. The doses used were 25 mg/d and 50 mg/d. Each mode of administration exhibited comparable efficacy, with the lower dose of 25 mg/d showing a favorable outcome (163).

According to the study by Freeman et al., the recurrence rate of PMS symptoms was significantly higher after short-term treatment compared to long-term treatment with sertraline. However, it should be noted that prolonged treatment also exhibited a high rate of symptom recurrence. Patients experiencing severe symptoms at the beginning of medication indicated the highest risk of relapse, regardless of the treatment duration. This study suggests that the high severity of complaints (before the start of treatment) is a marker for a worse prognosis in patients. The dosage used in this study ranged from 50 mg/d to 100 mg/d of sertraline (164).

On the other hand, the Yonkers et al. study shows no evidence of sertraline withdrawal symptoms after sudden discontinuation after 2 weeks of treatment for 2 cycles. This correlates with the theory cited above that short-term administration of sertraline is less likely to be fraught with side effects. The dose used ranged from 50 mg/d to 150 mg/d of sertraline, but the researchers did not consider the severity of initial symptoms (165).

According to a 2004 study, a dosing regimen (either continuous or luteal phase only) using 50 mg/d to 100 mg/d of sertraline does not show differences in efficacy for treating PMS/PMDD (151).

Due to the risk of relapse documented in the studies discussed above, this drug appears to be slightly inferior to fluoxetine. It should be noted that sertraline, in most studies, demonstrated efficacy in selected patients even at doses as low as 25 mg, emphasizing the necessity of individualizing therapy. Initiating therapy with a lower dose could potentially reduce symptoms and the risk of withdrawal syndrome, consequently lowering the risk of relapse, assuming its efficacy (**Table 3**). Moreover, sertraline has a shorter half-life than fluoxetine, which means it could be more convenient to use during luteal phase (166).

**Table 3:** Collected research on sertraline treatment in PMS.

Author, year of publication	Drug, dosage form and total daily dose	Study design	Dosage, total duration of administration	First test group		Second test group	Third test group	
Yonkers et al. 1997 (160)	**Sertraline hydrochloride** capsules 50 mg or 100 mg or 150 mg	1 cycle: single-blind, placebo-controlled3 cycles: randomized, double-blind,placebo-controlled	50 or 100 or 150 mg/day (If response was insufficient, dose was increased or matching placebo)during luteal phase	**Sertraline**121 women[24-45 years old](99 included in analysis)		**Placebo**122 women[24-45 years old](101 included in analysis)	–
Freeman et al. 1999 (161)	**Sertraline hydrochloride** capsules 50 mg or 100 mg or 150 mg	randomized, double-blind, placebo-controlled, parallel	50 or 100 or 150 mg/day (If response was insufficient, dose was increased) during 3 cycles	**Sertraline**62 women[18-45 years old]		**Desipramine hydrochloride** 50 women[18-45 years old]	**Placebo**55 women[18-45 years old]
Freeman et al. 2004 (151)	**Sertraline hydrochloride**tablets 50 mg or 100 mg	stratified, randomized, double-blind, placebo-controlled, parallel	50 or 100 mg/day (In the second or third cycle dose was increased to 100 mg) during 3 cycles (continuous or luteal phase dosing )	**Sertraline (Full- Cycle Dosing)** 56 women [18-45 years old](40 women included in analysis)		**Sertraline** (**Luteal- Phase Dosing)**56 women[18-45 years old](35 women included in analysis)	**Placebo**55 women[18-45 years old](43 women included in analysis)
Yonkers et al. 2015 (162)	**Sertraline hydrochloride**capsules 50 mg or 100 mg	double-blind, placebo-controlled, multisite,parallel-group, randomized, clinical	50 or 100 mg/day (If response was insufficient, dose was increased to 100 mg)from symptom-onset until the first few days of menses dosingduring 6 cycles	**Sertraline**125 women[18-40 years old]		**Placebo**127 women[18-40 years old]	–
Kornstein et al. 2006 (163)	**Sertraline hydrochloride** 25 mg or 50 mg	double-blind, placebo-controlled, randomized	25 or 50 mg/dayduring 4 cycles (luteal-phase and/or continuous and/or symptom-onset dosing)	**Sertraline** (25 mg)98 women[18-40 years old](74 women completed)		**Sertraline** (50 mg)97 women[18-40 years old](77 women completed)	**Placebo**101 women[18-40 years old](79 women completed)	
Freeman et al. 2009 (164)	**Sertraline hydrochloride** 50 mg or 100 mg	randomized, stratified, double-blind,placebo-controlled	50 or 100 mg/dayluteal-phase starting on day 14 before expected day of menses to menstrual day dosingduring 4 or 12 months (treatment group were studied for 18 months)	**Sertraline short-term** (drug for 4 months, placebo for 14 months) 87 women[18-45 years old](76 women were included in the analysis)		**Sertraline long-term** (drug for 12 months, placebo for 6 months) 87 women[18-45 years old](84 women were included in the analysis)	–	
Yonkers et al. 2005 (165)	**Sertraline hydrochloride** pill 50 mg or 100 mg or 150 mg	randomized, double-blind, placebo-controlled	50-150 mg/day (continuous dosing) or50-100 mg/day (luteal-phase starting on day 14 before expected day of menses dosing)during 3 cycles	**Sertraline (luteal- phase)**and placebo 281 women[24-45 years old]		**Sertraline (continuous)**and placebo 251 women[24-45 years old]	–
Author, year of publication	Method of data collection	Outcomes		Adverse events/ side effects		Limitations		Overall effect
Yonkers et al. 1997 (160)	The Daily Record of Severity of Problems,Hamilton Rating Scale for Depression, Clinical Global Impression Scale, Social Adjustment Scale	Participants taking sertraline experienced a significant decrease in their total daily symptom scores compared to those in the placebo group (depression, physical symptoms, and anger/irritability). The HDRS scores decrease in the sertraline group (44%) and in the placebo group (29%).		-adverse events occurred more frequently in the sertraline group than in the placebo group,-events occurring were: headache, nausea, insomnia, diarrhea, fatigue, and decreased libido		-the study cohort may not be generalizable to women with other psychiatric illnesses or to women with less severe PMS-regular monitoring through clinic visits and daily ratings could contribute to improved therapeutic response		positive
Freeman et al. 1999 (161)	Penn Daily Symptom Report (DSR), Hamilton Depression Rating Scale, Clinical Global Impressions–Severity Scale,Quality of Life Scale,Patient Global Ratings of Functioning and Improvement	Improvement were observed in all DSR factors with sertraline compared with desipramine and placebo, significantly for factors like mood and pain.DSR symptoms had decreased by more than 50% in 65% women in sertraline group, 36% in desipramine and 29% in placebo.		-adverse events occurred more frequently in the sertraline and desipramine group than in the placebo group,-sertraline resulted in a significantly higher incidence of nausea compared to the placebo group-conversely, dry mouth, dizziness, and constipation were significantly more frequent in the desipramine group compared to placebo.		-the study cohort may not be generalizable to women with other psychiatric illnesses or to women with less severe PMS		positive
Freeman et al. 2004 (151)	Daily Symptom Rating Form score and patient global ratings of functioning	Both groups of sertraline improved significantly more than the placebo group in DSRF scores (mood and physical symptoms were significantly more improved).Dosing with sertraline does not differ between continuous and premenstrual in PMS treatment.		-7 women from the full-cycle sertraline group, 5 women from the luteal-phase and 1 from placebo withdrawing from the study,-most frequent adverse events were gastrointestinal, decreased libido or orgasm, headache, insomnia, dry mouth, nausea, and nightmares		-the study cohort may not be generalizable to women with other psychiatric illnesses or to women with less severe PMS-small number of subjects with high postmenstrual symptom levels		positive
Yonkers et al. 2015 (162)	Premenstrual Tension Scale (PMTS) score,Inventory of Depressive Symptomatology–Clinician-Rated, Daily Record of Severity of Problems (DRSP),Clinical Global Impression (CGI) scales,Michelson SSRI (Selective SRI)Withdrawal Symptoms Scale scores	PMTS and CGI-S scores showed no significant differences.Symptom improvement was better with sertraline compared to placebo when measured by the IDS-C, CGI-I and DRSP		- nausea and difficulty sleeping occurred more frequently in the sertraline group		-maximum dose of 100 mg of sertraline hydrochloride-cohort size not meeting power estimates-attention and labor involved in charting symptoms may not be easily replicated in clinical settings		neutral/po sitive
Kornstein et al. 2006 (163)	Daily Symptom Report (DSR), Clinical Global Impressions- Severity of Illness and - Improvement scales,Patient Global Evaluation scale, Quality of Life Enjoyment and Satisfaction Questionnaire, Social Adjustment Scale-Self Report	Improvement were observed in total DSR scores for Intermittent luteal-phase sertraline dosing (25 mg/d - 50 mg/d) compared with placebo (across 2 cycles). Continuous and symptom-onset dosing were also effective (25 mg/d)		-most common adverse effects in all groups were: insomnia, nausea, headache		-homogeneity of the sample in terms of absence of medical and psychiatric comorbidity-short duration of the study-sequential design		positive
Freeman et al. 2009 (164)	total premenstrual DSR scores, Structured Clinical Interview, Hamilton Depression Rating Scale score,demographic variables	There were no significant differences in discontinuation rates between the two sertraline groups.Improvement was observed in 72% of patients in sertraline group.Patients in the short-term group and with low symptom severity were more likely to experience improvement in their symptoms.		-most frequent adverse effects were insomnia, nausea, fatigue, headache, decreased libido or orgasm and changes in appetite,		-definition of relapse is conservative in requiring that symptoms return to the study eligibility level and may underestimate the rate of relapse-results maynot be generalizable to all women with premenstrualsymptoms-the study did not explore alternative dosing strategies or investigate other treatment options for patients who relapsed or showed no improvement		positive
Yonkers et al. 2005 (165)	Daily Record of Severity of Problems (DRSP)	Administration of active medication during the luteal phase effectively reduced total DRSP scores, regardless of whether participants continued taking the medication throughout their entire cycle or stopped at the beginning of their period.		not mentioned		-researchers did not consider the severity of initial symptoms- the nine-item withdrawal factor has not been validated, and several key withdrawal symptoms were not ratedin the DRSP-withdrawal factor		positive

### 4.3 Paroxetine

A multicenter study using a placebo and paroxetine yielded a result indicating that paroxetine was effective in relieving PMDD symptoms. The study involved 327 women. Paroxetine was administered at doses of 12.5 mg/d or 25 mg/d or placebo once daily for three treatment cycles. The method for evaluating efficacy was the VAS-Mood score (focusing on symptoms such as irritability, tension, affective lability, and depressed mood) during the luteal phase. Both doses of paroxetine were found to be effective according to the VAS-Mood scale (167).

A clinical trial conducted by Landen et al. in 2006 demonstrated that continuous treatment of PMDD with paroxetine effectively reduced symptoms such as irritability, achieving a response rate of 85% compared to the placebo. Luteal-phase-only treatment showed comparable effectiveness to continuous administration for symptoms like irritability, affect lability, and mood swings. A modest effect on reducing the severity of symptoms was observed for depressed mood and somatic symptoms. Dosages ranging from 10 mg/d to 20 mg/d were utilized (168).

A 2008 study demonstrated the effectiveness of treating PMDD with paroxetine at a dose of 20 mg/d. The continuous treatment group achieved a response rate ranging from 50% to 78.6%, while the intermittent treatment group achieved a response rate ranging from 37.5% to 93.8%. The study was subject to limitations, including a small sample size of 36 participants and the absence of a placebo group (169).

In the Steiner et al., 2005 study, within the group using paroxetine during the luteal phase at a dose of 25 mg, at least one side effect was observed in 76.7% of patients. At a dose of 12.5 mg, it was observed in 67.7% of subjects. In the placebo trial, this percentage was 56.7%. The most commonly observed side effects associated with taking paroxetine were nausea, asthenia, headaches, and decreased libido (170).

According to the Landén et al., 2008 study, paroxetine demonstrates a rapid reduction in symptoms, which is uncommon for a serotonin-dependent antidepressant. Such a swift response to treatment has not been observed previously (146).

Paroxetine has shown high efficacy in treating premenstrual symptoms, although its use is associated with the frequent occurrence of at least one side effect (**Table 4**). It may emerge as an alternative to classical treatments for some patients due to its notably rapid action and very high efficacy.

**Table 4:** Collected research on paroxetine treatment in PMS.

Author, year of publication	Drug, dosage form and total daily dose	Study design	Dosage, total duration of administration	First test group	Second test group	Third test group	
Cohen et al. 2004 (167)	Paroxetine CR12.5 mg or 25 mg	multicenter, randomized, double-blind, placebo-controlled, fixed-dose	12.5 or 25 mg/day,during 3 cycles (continuously)	**Paroxetine** (12.5 mg)103 women[18-45 years old](70 women completed)	**Paroxetine** (25 mg)113 women[18-45 years old](72 women completed)	**Placebo**111 women [18-45 yearsold] (79 women completed)
Landén et al. 2007 (168)	Paroxetine capsules 10 mgor 20 mg	randomized, double-blind, placebo-controlled, 3 parallel groups	10 or 20 mg/day (firstand last 4 days was 10 mg)during 3 cycles (luteal- phase only or continuously)	**Paroxetine-continuous (PC)**60 women[≧̸18 years old](51 women completed)	**Paroxetine-intermittent (PI)**59 women[≧̸18 years old](55 women completed)	**Placebo (PBO)**59 women[≧̸18 years old](51 women completed)
Wu et al. 2008 (169)	Paroxetine capsules 20 mg	randomized, controlled, open-label, parallel design, non-blinded, prospective	20 mg/dayfor 2 cycles(continuously), then during 4 cycles (luteal-phase only or continuously)	**Paroxetine-continuous** (first 2 cycles)36 women[18-45 years old]	**Paroxetine-continuous** (4 cycles)14 women[18-45 years old]	**Paroxetine-intermittent**(4 cycles)16 women[18-45 years old]
Steiner et al. 2005 (170)	Paroxetine CR12.5 mg or 25 mg	multicenter, randomized, double-blind, placebo-controlled, fixed-dose	12.5 or 25 mg/dayfor up to 3 cycles (luteal- phase)	**Paroxetine** (12.5 mg)130 women[18-45 years old](104 women completed)	**Paroxetine** (25 mg)116 women[18-45 years old](87 women completed)	**Placebo**120 women[18-45 years old](101 completed)
Landén et al. 2009 (146)	Paroxetine capsules 20 mg	randomized, double-blind, placebo-controlled, crossover trial	20 mg/dayfrom date around ovulation to 3 day of menstruationduring 3 cycles (one of three cycles was alwaysplacebo)	**Placebo -> Paroxetine -> Paroxetine**8 women[≧̸18 years old](7 women completed)	**Paroxetine -> Placebo -> Paroxetine**7 women[≧̸18 years old](7 women completed)	**Paroxetine -> Paroxetine -> Placebo** 7 women[≧̸18 years old](7 women completed)
Author, year of publication		Method of data collection	Outcomes		Adverse events/ side effects	Limitations	Overall effect
Cohen et al. 2004 (167)		Visual Analogue Scale-Mood (irritability, tension, affective, CGI-I lability, depressed mood) score	Both groups of paroxetine showed significant improvement (also for physical symptoms and social functioning) in VAS-Mood scores compared to placebo group.		-adverse events occurred in all groups, but to the least extent in placebo,-most frequent adverse effects were asthenia, libido decreased and female genital disorders	-short duration of the study- the primary outcome measure was patient-rated	positive
Landén et al. 2007 (168)		VAS and PMTS-O scales, CGI-I and PGE scales	Both groups of paroxetine showed significant improvement (significantly in irritability, affect lability, mood swings, depressed mood and tension) in VAS-Mood scores compared to placebo group. Intermittent treatment was as effective as continuoustreatment for certain symptoms.		-nausea, headache, somnolence and more side effects occurred in all groups	- VAS-rated symptoms other than irritability were not defined as primary effect parameters-short duration of the study	positive
Wu et al. 2008 (169)		Prospective Record of the Impact and Severity of Menstrual Symptomatology (PRISM) calendar,HAMD, HAMA, Zung-SDS, STAI, and CGI-S	Paroxetine was effective in improving symptoms of PMDD in both treatment groups, with response rates varying between the two groups, and the effects lasting for six consecutive menstrual cycles.		not mentioned	-open-label design-lack of placebo group-small sample size	positive
Steiner et al. 2005 (170)		observer-rated PremenstrualTension Scale (PMTS-O), global assessment of disease severity (CGI-S, Severity of Illness item),global assessment of disease improvement (CGI-CI, Global Improvement item),patient global evaluation (PGE),patient-rated assessment of	Paroxetine was effective in treating PMDD symptoms at both groups. Patients taking paroxetine CR showed significant improvement in both the main measure of effectiveness and in additional symptom measures compared to those taking a placebo.		-adverse event occured in all groups,-most frequent adverse effects were nausea, asthenia, headache and libido decreased	-lack of a direct comparison study between continuous and intermittent administration of paroxetine CR-short duration of the study-The study did not explore the long-term effects or efficacy of luteal phase dosing with paroxetine CR	positive
Landén et al. 2009 (146)		self-rated irritability using a VAS,serum levels of paroxetine, patient-rated CGI-I	Paroxetine was found to be effective in reducing irritability.		- in the first paroxetine treatment cycle, 68.2% reported experiencing nausea at least once.-4 hours after taking medication/placebo the difference in the number of subjects experiencing nauseareached statistical significance	-small sample size-considerable number of nonresponders-short duration of the study-only one adverse effect studied-inability to draw conclusions about the feasibility of symptom-onset dosing for premenstrual symptomsother than irritability	positive

### 4.4 Escitalopram

A study by Eriksson on the efficacy of escitalopram suggests a higher effectiveness of this drug than a placebo. The study involved 151 women, and the drug was administered intermittently for 3 months, only during the luteal phase. The doses used were 10 mg/d and 20 mg/d. The use of the 20 mg/d dose showed a symptom-reducing effect of up to 90%. The primary measurements focused on the sum of symptoms such as irritability, depressed mood, tension, and affective lability. Irritability alone, considered the main symptom of PMDD in this study, was reduced by 80% compared to the placebo group — a reduction of 30%. Side effects, such as nausea and reduced libido, were not observed more frequently in patients receiving escitalopram at 20 mg/d than in those receiving a lower dose (46, 171).

The Freeman et al., 2005 study also demonstrated the efficacy of treating PMDD with escitalopram at doses ranging from 10 mg/d to 20 mg/d. However, the study was constrained by limiting factors, such as a low number of participants and the absence of a placebo trial (172).

Escitalopram, due to the limited number of available studies, cannot be conclusively evaluated as an effective drug for the treatment of PMS (**Table 5**).

**Table 5:** Collected research on escitalopram treatment in PMS.

Author, year of publication	Drug, dosage form and total daily dose	Study design	Dosage, total duration of administration	First test group	Second test group	Third test group
Eriksson et al. 2008 (171)	Escitalopramtablet (as the oxalate salt) 10 mg or 20 mg	randomized, parallel-group, placebo- controlled, double-blind, single-center	10 or 20 mg/day,for 3 cycles (intermittently during luteal phases)	**Escitalopram 10 mg**(first 2 days with 5 mg)54 women[≧̸18 years old] (50 women wereincluded in analysis)	**Escitalopram 20 mg**(first day 5 mg, second day 10 mg)53 women[≧̸18 years old](51 women were included in analysis)	**Placebo**51 women[≧̸18 years old](50 women were included in analysis)
Freeman et al. 2005 (172)	Escitalopram 10 mg or 20 mg	randomized, double-blind, preliminary	11 or 20 mg/dayfor 3 cycles (during luteal phases or symptom-onset)	**Escitalopram** luteal- phase13 women[≧̸18 years old]	**Escitalopram** symptom-onset (first 2 days with 5 mg)14 women[≧̸18 years old]	–
Author, year of publication	Method of data collection		Outcomes	Adverse events/ side effects	Limitations	Overall effect
Eriksson et al. 2008 (171)	self-assessed VAS,10-item Premenstrual Tension Syndrome Scale– Observer Rating (PMTS-O),Sheehan Disability Scale,Clinical Global Impression–Severity (CGI-S) scale, Clinical Global Impression–Improvement (CGI-I) scale, Patient Global Evaluation (PGE)(SDS)		reduction of irritability, depressed mood, tension, and affective lability with 20 mg/day dose	the most frequent adverse effects were nausea, fatigue, dry mouth and decrease in sexual interest in all groups	-high placebo response rate-short duration of the study-lack of comparison with continuous treatment-no exploration of long-term effects or relapse rates	positive
Freeman et al. 2005 (172)	17-item Penn Daily Symptom Report (DSR), Clinical Global Impressions-Improvement scale, Hamilton Rating Scale for Depression, Sheehan Disability Scale		a significant improvement of DSR score in both groups,a significant clinical improvement in both groups	Escitalopram was associated with good tolerability. Side effects were minor and short- lived, with only two participants discontinuing treatment due to medication-relatedadverse events	-small sample size-short duration of the study-lack of placebo group	positive

### 4.5 Citalopram

In a 1998 study by Wikander et al., examining how the use of citalopram affects the treatment of premenstrual dysphoria with severe irritability, pharmacological medication was shown to be more effective than placebo. The drugs were administered either continuously or during the luteal phase only. The study revealed that the administration of the 20 mg drug during the luteal phase alone led to better control of irritability and improvement in well-being compared to continuous use of the drug (173).

Another study, in turn, demonstrated that citalopram administered as needed, in doses ranging from 10 mg/d to 20 mg/d, also showed efficacy in relieving PMDD symptoms (174).

On the other hand, Freeman et al. suggest that the treatment of PMS with citalopram is effective for patients in whom prior treatment with SSRIs has failed, whether used throughout the entire menstrual cycle or only during the luteal phase (175).

Studies on the treatment of PMS or PMDD with citalopram are riddled with limitations, including small subject numbers and insufficient independent research, hindering a comprehensive evaluation of citalopram’s efficacy in PMS treatment (**Table 6**). Most studies underscore the effectiveness of citalopram when used intermittently—specifically, during the luteal phase of the monthly cycle.

**Table 6:** Collected research on citalopram treatment in PMS.

Author,year of publication	Drug, dosage form and total daily dose	Study design	Dosage, total duration of administration	First test group	Second test group	Third test group	Fourth test group
Wikander et al. 1998 (173)	Citalopramcapsule 10 mg or 20 mg or 30 mg	randomized, double-blind, placebo-controlledwith 4 parallel groups	10mg or 20mg or 30 mg/day (based on side effects and symptom improvement, base dose-20 mg)during 3 cycles (continuous or semi-intermittent or intermittent)	**Citalopram continuous (CC)**19 women [≧̸18 years old](17 women included in analysis)	**Citalopram semi- intermittent (CS)**20 women[≧̸18 years old] (17 women included in analysis)	**Citalopram Intermittent (CI)**19 women [≧̸18 years old](18 women included in analysis)	**Placebo (PL)**20 women[≧̸18 years old] (17 women included in analysis)
Ravindran et al. 2007 (174)	Citalopram10 mg or 20 mg	single-center open naturalistic flexible-dose	10 or 20 mg/dayduring 2 cycles	**Citalopram** symptom- onset7 women [18-45 years old](6 women completed)	–	–	–
Freeman et al. 2002 (175)	Citalopram20 mg or 40 mg	randomized naturalistic open-label	20 or 40 mg/dayduring 3 cycles (half or full)	**Citalopram** (half-cycle, 20-40 mg)11 women[18-45 years old]	**Citalopram** (full-cycle 10-20 mg)6 women [18-45 years old]	–	–
Author, year of publication	Method of data collection	Outcomes		Adverse events/ side effects		Limitations	Overall effect
Wikander et al. 1998 (173)	Daily symptom self-ratings using VAS,measurement of serum concentrations of citalopram, intent-to-treat analysis	significant improvement in the CC and CI groups over the PL group,the global self-rating seemed better in the CI group than in the CI and the CS groups		Few participants stopped treatment due to side effects: headache (1 in CC, 1 in CI), sedation (2 in CS, 1 each in CC, PL), and anxiety/tension (1 in CC, 1 in PL), side effects being uncommon, mild, and short- lived.Reduced sex drive, dry mouth, and sweating were the most frequently reported issues.		-small sample size-short duration of the study-flexibility in dosage may impact the expected relationship between dose intake and serum levels	positive
Ravindran et al. 2007 (174)	Premenstrual tension scale (PMTS-O),Clinical Global Impression of Improvement (CGI)	symptoms improved significantly compared to before treatment,significant improvement in the CGI scale and PMTS-O scores in both cycles		3 participants taking the 20 mg dose experienced restlessness, dizziness, nausea, and diarrhea.1 person on the 10 mg dose reported mild anxiety and restlessness.		-small sample size-relatively brief duration of follow-up-short duration of the study-lack of placebo group	positive
Freeman et al. 2002 (175)	Total premenstrual Daily Symptom Report (DSR) scores, Hamilton Depression Rating Scale (HAM-D-29)	significant improvement in total DSR scores and HAM-D-29 scores in both (half-cycle and full-cycle) treatment groups		12 participants during the first cycle reported nausea, insomnia, dry mouth, and general digestive issues (each reported by 4 participants),the occurrence of side effects did not differbetween the half-cycle (64%) and full-cycle (63%) dosing groups.		-small study group-uncontrolled and unblinded trial design-previous SSRI failure determined in uncontrolledconditions	positive

### 4.6 Venlafaxine

A randomized controlled double-blind clinical trial evaluating the efficacy of venlafaxine as a representative of the SNRI group demonstrated its significant superiority over placebo in reducing PMDD symptoms. The study included 143 women who were administered venlafaxine for four menstrual cycles at doses ranging from 50 to 200 mg/d. In the group receiving the drug, 60% of patients experienced symptom relief, compared to 35% in the placebo group (176).

A study by Cohen et al. suggests that venlafaxine is effective and well-tolerated in the treatment of PMDD at doses ranging from 75 mg/d to 112.5 mg/d. However, this open-label study is significantly limited by a small sample size (177).

The study by Hsiao et al. also indicates the efficacy of venlafaxine in the treatment of PMDD. Patients reported relief from symptoms such as anxiety and depression. Doses ranging from 18.25 mg/d to 150 mg/d were used. However, the study was limited by a small number of participants and excessive variability in the doses administered, which were modified by the patients themselves (178).

Venlafaxine appears to be effective in treating premenstrual symptoms such as anxiety and depression, but the limited number of studies is a significant drawback, preventing a comprehensive evaluation of the drug’s effectiveness. It should be noted that the effect of venlafaxine at the doses used in the studies primarily corresponds to an enhancement of serotonergic rather than noradrenergic conduction. It is plausible to consider the use of this drug in cases of high intolerance to SSRI drugs as an alternative in the treatment of PMS/PMDD.

### 4.7 Duloxetine

Duloxetine is a medication that is not only used in the treatment of psychiatric disorders but is also indicated for alleviating painful physical symptoms that may accompany depression (179). This suggests, in combination with its serotonergic component, that the drug could be effective for PMS associated with increased pain.

Ramos et al. present two female patients suffering from PMDD. In the case of one patient who had an isolated premenstrual disorder, there was an improvement of up to 94% in the premenstrual DRSP score with a daily dose of 60 mg. On the other hand, the second patient, being treated for Major Depressive Disorder (MDD), continued to experience severe mental symptoms despite previous psychiatric treatment, including venlafaxine 375 mg/d and clomipramine 150 mg/d. After the administration of 120 mg/d duloxetine, not only was there a satisfactory control of depression observed but, notably, the patient did not manifest premenstrual symptoms for the first time (180).

In contrast, a study by Mazza et al. indicated that duloxetine at a dose of 60 mg caused a significant improvement in symptoms (50% improvement) in almost 80% of patients. However, this study did not include a placebo group. Significantly notable was the elevated rate of improvement within a brief timeframe—following the initial two cycles during which the patients underwent drug administration (181). Another single-blind study demonstrated a swift clinical response in female patients, manifesting as early as the first cycle when duloxetine was administered at a dosage of 60 mg (182).

Duloxetine is a drug characterized by a dual mechanism of action that is particularly beneficial in the context of heightened physical pain. It is feasible to administer a relatively low dose of 60 mg/d. Adverse effects were observed in a small percentage of subjects, encompassing symptoms such as nausea, insomnia, decreased libido, and reduced appetite. However, it is crucial to acknowledge that studies evaluating this substance are hampered by significant limitations, including the absence of double-blinding, a limited cohort of female patients, or the complete absence of a placebo group. Further investigations employing double-blind, placebo-controlled trials are imperative to advance our understanding.

### 4.8 Buspirone

Buspirone affects serotonergic conduction through the 5HT1A receptor and also has properties that affect dopaminergic pathways (183). Both of these actions suggest a potential use of the drug in the treatment of PMS and PMDD.

In a single-blind study, Nazari et al. compared buspirone 10 mg/d with fluoxetine 20 mg/d, demonstrating that both formulations were effective with no advantage for either of them. However, it is conceivable that buspirone, due to its lower rate of side effects, may be the preferred drug to fluoxetine (154). Nevertheless, the study lacked a control group and had a short duration of 2 months. Conversely, another study comparing buspirone and nefazodone found that buspirone showed a better treatment effect than placebo, in contrast to nefazodone (184).

The limited number of studies and the absence of double-blind trials constrain the robustness of utilizing this substance. However, it appears to be effective and may be considered an option for certain patients.

### 4.9 Conclusion

In conclusion, SSRI drugs are highly effective in treating PMS and PMDD, particularly the irritability associated with the syndrome. Their use is linked to relatively mild side effects, which can be mitigated by employing the drugs intermittently. These characteristics justify their use as first-line drugs in the treatment of PMS. Fluoxetine demonstrates significant therapeutic efficacy and induces relatively few side effects at therapeutic doses in a luteal-phase-only regimen. Moreover, fluoxetine has undergone extensive study for PMS treatment, making it the most suitable drug for managing the disorder. Paroxetine also exhibits high efficacy in treating PMS, though its elevated rate of side effects renders it less preferable compared to fluoxetine.

Sertraline, due to its high rate of symptom recurrence, does not appear to be the best drug for treating premenstrual symptoms. Assessing the actual effectiveness of escitalopram, citalopram, and venlafaxine in the treatment of PMS is challenging due to the small number of studies and the limited number of participants. However, the selection of a particular SSRI drug should be based on individual patient preference and adjusted for efficacy and tolerability, as mentioned by the authors of the 2013 Marjoribanks et al. review (46). The treatment regimen should also be based on the patient’s needs. While it can be assumed that other SSRI drugs are also effective, they may differ in the occurrence of side effects. It is presumed that all doses used in the cited studies are effective in treating PMS. However, there is a correlation indicating that the incidence of SSRI side effects increases with dosage, making high doses potentially intolerable for patients. It is noteworthy that a significant number of patients with PMS were administered doses that were akin to those utilized in the treatment of affective disorders. Doses developed theoretically for a more severe disorder and on groups, typically not including women, can often be too high. This is worth bearing in mind, as individual studies using lower doses in selected cases have proven effective and reduced the risk of side effects.

## 5 Herbal treatment

Most of the herbal research is fraught with significant limitations, constraining the ability to arrive at conclusive evaluations (**Table 7**). The predominant focus in existing studies centers around VAC, saffron, and curcumin.

**Table 7:** Collected research on selected herbs in PMS.

Author,year of publication	Herb, dosageform and total daily dose	Study design	Dosage,total duration of administration	First test group	Second test group
Ozgoli et al.2009 (185)	***Ginkgo biloba L.*** coated tablets, 120 mg	randomized, single-blind, placebo-controlled	3 x 40 mg/dayfrom day 16 of the cycle to day 5 of the next cycle, during 2 menstrual cycles	**Ginkgo tablets**45 women [18-30 years old](43 women were included in the analysis)	**Placebo**45 women [18-30 years old](42 women were included in the analysis)	
Sharifi et al.2014 (186)	**Chamomile *(Matricaria chamomila)*** capsules, 300 mg	prospective, randomized, double blind	3 x 100 mg/dayfor 7 days,during 2 menstrual cycles	**Chamomile capsules**59 women [18-35 years old](45 women were included in the analysis)	**Mefenamic acid capsules (MA), 250 mg**59 women [18-35 years old](45 women were included in the analysis)	
Yamada and Kanba 2007 (187)	**Tsumura kampo medicine, kamishoyosan (TJ- 24)**extract granules 7,5 g	open-labeled pilot study	3 x 2.5 g/day during 6 menstrual cycles	30 women [18-48 years old](26 women were included in the analysis)	–	
Jung-Gum et al.2010 (188)	**St. John's wort (SJW) (Hypericum perforatum)**tablets 600 mg	randomized double-blind placebo-controlled	2 x 300 mg/day during 2 menstrual cycles(6 weeks)	**SJW**25 women [20-30 years old]	**placebo**26 women [20-30 years old]	
Canning et al.2010 (189)	**St John’s Wort (Hypericum perforatum)**coated tablets 900 mg	randomized, double-blind, placebo-controlled, crossover	2 x 450 mg/day during 2 menstrual cycles	**Hypericum perforatum (2 cykle) → washout (1 cykl) → placebo (2 cykle)** 19 women [18-45 years old](17 women were included in the analysis)	**placebo (2 cykle) → washout (1 cykl) → Hypericum perforatum (2 cykle)**17 women [18-45 years old](15 women were included in the analysis)	
Stevinson and Ernst 2000 (190)	**St John’s Wort *(Hypericum perforatum)*** tablets 300 mg(standardised to 900µg hypericin)	prospective, open, uncontrolled, observational pilot study	1 x 300 mg/day during 2 menstrual cycles	25 women [18-50 years old](19 women were included in the analysis)	–	
Farahmand et al.2020 (191)	**Anise (*Pimpinella Anisum*)**capsules 330 mg	randomized double-blind placebo, controlled	3 x 110mg/dayfrom day 21 of the cycle to day 3 of the next cycle (10 days in total) during 2 menstrual cycles	**Anise capsules**42 women [18-35 years old] (35 women were included in the analysis)	**placebo**42 women [18-35 years old] (32 women were included in the analysis)	
Farahmand et al.2020 (192)	**Echium amoenum (EA)**capsules 450 mg	randomized double-blind placebo, controlled	3 x 150mg/dayfrom day 21 of the cycle to day 3 of the next cycle (10 days in total) during 2 menstrual cycles	**EA capsules**42 women [18-35 years old] (37 women were included in the analysis)	**placebo**42 women [18-35 years old] (32 women were included in the analysis)
Khayat et al.2014 (193)	**Ginger (Zingiber officinale)** capsules 500 mg	randomized double-blind placebo-controlled	2 x 250mg/dayfrom day 21 of the cycle to day 3 of the next cycleduring 3 menstrual cycles	**Ginger**35 women [18-35 years old] (33 women were included in the analysis)	**placebo**35 women [18-35 years old] (33 women were included in the analysis)
Akbarzadeh et al.2015 (194)	**Melissa (Melissa officinalis)** capsules 1200 mg	randomized double-blind placebo-controlled	2 x 600mg/day during 3 menstrual cycles	**melissa**50 women [average age - 16 years] (50 women were included in the analysis)	**placebo**50 women [average age - 16 years] (50 women were included in the analysis)
Tjandrawinata et al.2011 (195)	**bioactive extract of Phaleria macrocarpa (DLBS1442)**capsules 200-300 mg	open study	2 or 3 x 100 mg/dayfrom the 3rd last day of the cycle to the first 3rd day of the following cycle (average 6 days)during 4 menstrual cycles	**placebo (2 cykle) → DLBS1442 (2 cykle)**23 women [20-40 years old]	–
Sodouri et al.2013 (196)	**Zataria Multiflora (ZM)**pearls 80 mg	randomized double-blinded prospective	4 x 20mg/day7 days before first day of cycle during 2 menstrual cycles	**ZM**44 women [18-35 years old] (38 women were included in theanalysis)	**placebo**44 women [18-35 years old] (37 women were included in theanalysis)
Author, year of publication	Method of data collection	Outcomes	Adverse events/ side effects	Limitations	Overall effect
Ozgoli et al.2009 (185)	a self-administered questionnaire about daily symptom rating	Reduced severity of sleep disturbances, fatigue, bloating and palpitations only in the Ginkgo group. Reduce the severity of most other physical and psychiatric symptoms to a greater extent in the Ginkgo group than in the placebo group.	-nausea (1 person in the Ginkgo group and 4 in the placebo group)-increased desire for sleep (2 people in the Ginkgo group)	-small sample size-participants in the study were only female students, which does not reflect the general population of women with PMS-women were only observed for 2 menstrual cycless while taking Ginkgo/placebo, making it impossible to assess the long-term effects of Ginkgo on PMS symptoms-not very objective method of data collection through self- completion of questionnaires by participants-the placebo tablets were made of starch, which may have affected some PMS symptoms	positive
Sharifi et al.2014 (186)	the questionnaires on the efficacy, side effect of the capsules and satisfaction with treatment	Chamomile capsules reduced the overall intensity of PMS symptoms, mainly psychological symptoms (anger and irritability) from MA	-more severe menstrual bleeding (in the Chamomile group)-more severe GI complications (in the MA group)	-small sample size-participants in the study were only female students, which does not reflect the general population of women with PMS-women were only observed for 2 menstrual cycles while taking Chamomile/MA, making it impossible to assess the long-term effects of Chamomile on PMS symptoms-not very objective method of data collection through self- completion of questionnaires by participants	positive
Yamada and Kanba 2007 (187)	The scores of the Global Assessment of Functioning (GAF) Scale and Hamilton Depression Rating (HAM- D) Scale in the late luteal phase	GAF and HAM-D total scores improved significantly after the treatment.14 patients (46.7%) hadremission, 5 (16.7%) showed improvement.	-hot flushes (1 person who could not continue TJ-24, not included in the analysis)	-small sample size-no placebo group-need to conduct a larger, well-controlled, double-blind, placebo- controlled study to confirm results-whether PMS symptoms can be improved after a shorter time of use of SJW (e.g. after 2 menstrual cycles) has not been tested	propably positive
Jung-Gum et al.2010 (188)	The questionnaire, which contained Beck Depression Inventory (BDI), Visual Analogue Scale (VAS, and Premenstrual Assessment Form (PAF) filled before and after treatmentThe menstrual daily diary for PMS (supplemented the PAF) to record daily symptoms by participants (during the experiment,around 3 or 4 months)	No significant changes in total BDI, VAS or PAF scores were observed.Improved score in the SJW group regarding emotional lability, hostility/anger and impulsivity compared to the placebo group.	-nausea (four women, but only on the first day)	-small sample size-women were only observed for 2 menstrual cycles while taking St. John's wort/placebo, making it impossible to assess the long- term effects of St. John's wort on PMS symptoms-narrow age range of women (20-30 years), which does not reflect the general population of women with PMS	neutral/ positive
Canning et al.2010 (189)	the Diary booklets which include:-the Daily Symptom Report (DSR), a checklist-the State scale of the State-Trait Anxiety Inventory (STAIS), BDI, Aggression Questionnaire (BPAQ) and Barratt Impulsiveness Scale version 11 (BIS-11)Biochemical Measures: the hormone (estradiol, progesterone, testosterone, LH, FSH and prolactin) and cytokine (IL-1b, IL-6, IL-8, IFNg and TNFa)	Significant impact on physical (appetite, swelling) and behavioural (poor coordination, insomnia, confusion, headaches, crying, fatigue) symptoms of PMS in the Hypericum group in both goups. No significant changes in PMS symptoms related to pain or mood were noticed (pain symptoms only seemed to decrease towards the end of the treatment period).no significant differences in biochemical measures	-digestive and respiratory symptoms (almost equally distributed between both groups)	-small sample size-not very objective method of data collection through self-completion of questionnaires by participants	positive
Stevinson and Ernst 2000 (190)	The Daily Symptom Ratings (DSR), a checklistthe modified, self-report Social Adjustment Scale (SAS-M), the Hospital Anxiety and Depression scale (HAD)	improving the total score of SAS-M and HAD scale	nausea, constipation, flatulence, dizziness, heavy menstrual flow (5 women, at the beggining of study)	-small sample size-no placebo group-women were only observed for 2 menstrual cycles while taking St. John's wort, making it impossible to assess the long-term effects of St. John's wort on PMS symptoms-a randomised, placebo-controlled, double- blind study should be conducted to confirm the results	propably positive
Farahmand et al.2020 (191)	Premenstrual Symptoms Screening Tools (PSST) questionnaire	Significantly reduce PMS symptoms and improved PSST total score in the Anise group.	-nausea (1 case) and menorrhagia (1 case) in the Anise group-nausea (2 cases) and diarrhea (1 case) in the placebo group	-small sample size-participants in the study were only collage students, which does not reflect the general population of women with PMS-not very objective method of data collection through self-completion of questionnaires by participants-Anise was just prescribed for 2 menstruation cycles, making it impossible to assess the long- term effects	positive
Farahmand et al.2020 (192)	Premenstrual Symptoms Screening Tools (PSST) questionnaire	effective reduce of PMS symptoms, mainly anxiety/tension and crying symptoms and improved Premenstrual Symptoms Screening Tool (PSST) total score in the EA group.	-nausea (3 cases) in the placebo group	-small sample size-participants in the study were only female students, which does not reflect the general population of women with PMS-EA was just prescribed for two menstruation cycles, making it impossible to assess the long- term effects-not very objective method of data collectionthrough self-completion of questionnaires by	positive
Khayat et al.2014 (193)	the daily record questionnaire	reduction in the severity of mood and physical and behavioural symptoms in the ginger group	-nausea in the group of ginger	-small sample size-participants in the study were only female students, which does not reflect the general population of women with PMS-ginger was just prescribed for three menstruation cycles, making it impossible to assess the long-term effects-not very objective method of data collection through self-completion of questionnaires by participants	positive
Akbarzadeh et al.2015 (194)	Premenstrual Symptoms Screening Tools (PSST) questionnaire	reduction in physical, psychological and social PMS symptoms and improvement in total PSST scores in the melissa group	not mentioned	-small sample size-participants in the study were only high school girls, who were at different stages of puberty, which does not reflect the general population of women with PMS-not very objective method of data collection through self-completion of questionnaires by participants-high placebo effect	positive
Tjandrawinata et al.2011 (195)	Daily self-assessment using a visual analog scale (VAS) in a symptom diary	noticeable relief of PMS pain symptoms, including but not limited to abdominal pain, back pain and fatigue and improvement of VAS total score	few adverse events reported, mostly mild in severity, the most commonly reported adverse events were:-diarrhea (6 reports)	-small sample size-no comparison group-need to conduct a larger, additionally randomised with placebo group study to confirm results	propably positive
Sodouri et al.2013 (196)	prospective record of the impact and severity of menstrual symptoms (PRISM) in the form of a calendar.	No change in severity or incidence of symptoms was noted between the ZM group and placebo.	not mentioned	-small sample size-participants in the study were only collage students, which does not reflect the general population of women with PMS-ZM was just prescribed for 2 menstruation cycles, making it impossible to assess the long-term effects-high placebo effect-the effects of other dosages need furtherinvestigation, cause this dosage might could not control the symptoms of PMS	neutral

### 5.1 Chasteberry’s extract (Vitex Agnus-Castus, VAC)

VAC is a herbal preparation, and its mechanism of action primarily involves enhancing dopaminergic conduction (197). An empirical argument supporting the use of a drug with this affinity is the frequent occurrence of hyperprolactinemia in women experiencing some symptoms of PMS (198).

In his study, Schellenberg demonstrated that, over time, the number of patients responding to VAC treatment increased. By the study’s conclusion, which encompassed three cycles, more than half of the women taking VAC experienced a symptom reduction of over 50%. In contrast, the placebo group exhibited a reduction of only 24%. Another study by Schellenberg et al. revealed that the most optimal therapeutic effects were achieved with a 20 mg dose taken once daily, with no additional benefits observed when increasing the dose to 30 mg (199).

Furthermore, according to Cerqueira et al., VAC demonstrated improvements in both physical and psychological symptoms of PMS and PMDD (200). Another study also underscored the efficacy of VAC extract, indicating enhancements in all PMS symptom domains as measured by PMSD, except for abdominal cramping (201). It is worth noting, however, that abdominal cramping may be inherent to the nature of PMS.

In contrast, Van Die et al. suggested the potential use of VAC in combination with St. John’s wort for premenopausal women with PMS. The results showed promise, especially in addressing mood swings. Despite the observation of improvements in anxiety and hyperhydration levels, the degree of enchantment was not significantly different from that observed with a placebo. It is essential to note that this study was conducted with a small group (14 people) (202).

Moreover, Ma et al. highlighted the efficacy of the substance in controlling symptoms related to water retention in a study with a larger participant pool (203). A noteworthy concurrence with this study is the observed progressive improvements over time, which align with findings in the earlier mentioned Schellenberg study. Interestingly, the study also reported a relatively high percentage of placebo results, potentially attributed to the subjective measurement methods employed. This methodology may explain the results in the He et al. study, where VAC demonstrated improvement but placebo results were as high as 50% (204).

Ambrosini et al. supported the effectiveness of VAC in controlling PMS-related headaches (205). Presumably, the herb’s impact on headaches is linked to its high affinity for μ and κ subtype opioid receptors (197). However, it is essential to note that the study lacked a control sample. Additionally, the Bergel et al. study observed that persistent headaches associated with VAC led one patient to discontinue treatment. Interestingly, in this study, VAC did not demonstrate an impact on prolactin levels (206).

This raises questions about the credibility of the previously mentioned study, particularly since this study, revealing headaches as a side effect despite high limitations, provides more qualitative data than the research discussed earlier. On the other hand, He et al. whose study also identified headaches after VAC administration, suggested that many reported side effects might be attributed to the inherent nature of PMS (204).

Most of the cited studies face significant limitations, a point emphasized by Verkaik et al. In their meta-analysis on VAC studies in PMS, they highlighted the high risk of bias, heterogeneity, subjective methods, and underpowered inclusion criteria, collectively diminishing the quality of evidence regarding the effectiveness of VAC (207). More discerning and selective studies are imperative. Furthermore, many herbal studies exhibit notable methodological flaws, such as the absence of a placebo group.

In conclusion, while VAC appears to be an effective formulation, further research is essential to conclusively demonstrate its benefits. It is important to keep in mind that herbal therapies are frequently associated with various unpredictable interactions, potentially contributing to the high number of side effects, as seen in the Berg et al. study where the group was allowed to take other drugs in addition to VAC (206).

### 5.2 Saffron

The argument in favor of using saffron finds support in studies on the substance conducted in affective disorders (208). In rat studies, saffron has been shown to increase BDNF expression (209) in the hippocampus. The suspected mechanism of action involves the safranal and crocin compounds, which impact serotonergic conduction (210). Additionally, this preparation contains flavonoids and carotenoids, which exhibit antioxidant effects and prevent the formation of prostaglandins, potentially explaining its analgesic effect (211).

Conversely, a study by Fukui et al. discovered that a 20-minute exposure to the scent of the preparation lowered cortisol levels and elevated estrogen levels, irrespective of the menstrual cycle timing. This exposure was associated with symptom relief, as measured by the STAI (State-Trait Anxiety Inventory). It is worth noting that the rapid decrease in cortisol levels may indicate a beneficial short-term effect of saffron exposure in situations of heightened tension and stress (212).

In the study by Beiranvand et al., saffron was administered once a day for two menstrual cycles at a dose of 30 mg, revealing a significant decrease in PMS severity in the saffron group compared to the placebo group (213). This finding aligns with an earlier study by Agha-Hosseini et al., where saffron, at a total dose of 30 mg divided into two doses of 15 mg each, demonstrated a notable reduction in PMS symptoms for up to 76% of women after two monthly cycles of administration (214).

Rajabi et al. explored the impact of saffron on PMDD symptoms by comparing it with an antidepressant and a placebo, similar to previous studies on depression. Both preparations were administered twice a day. The dosage was limited to the luteal phase based on the mechanism of action. The results indicated comparable efficacy between fluoxetine and saffron (215). Notably, side effects were less frequently observed with saffron, potentially favoring the herbal preparation.

Saffron appears to offer an alternative to SSRI drug treatment for PMS. However, further studies are warranted to both indicate and confirm its effectiveness.

### 5.3 Curcumin

Curcumin, a member of the ginger family, is a curcuminoid derived from turmeric. Its suspected mechanism of action in addressing PMS is linked to the modulation of neurotransmitter levels, including serotonin (216). Additionally, a study by Fanaei et al. demonstrated an increase in BDNF levels in women with PMS following curcumin supplementation, which correlated with clinical improvement in patients (217). Another pivotal aspect of curcumin’s action involves the inhibition of prostaglandin synthesis by suppressing COX-2 (218, 219).

Khayat et al. demonstrated that administering curcumin for 10 days starting 7 days before menstruation is an effective method for relieving PMS symptoms compared to a placebo (220). They utilized a dose of 100 mg twice daily. On the contrary, Bahrami et al. used a higher dose of 500 mg once a day with the same dosing schedule but found no clear advantage of curcumin over placebo (221). However, both studies are limited by the relatively small number of subjects and the short study duration of 3 cycles.

Another aspect of curcumin’s impact on women with PMS was explored by Arabnezhad et al., who studied vitamin D levels using a 500 mg dose of curcuminoid and 5 mg of piperine following the same schedule. They reported a slight improvement in vitamin D levels relative to the placebo. On the contrary, the markers of liver and kidney function measured in this study did not exhibit differences between the study group and the placebo (222). Another study analyzed the effects of curcuminoid and piperine on inflammatory markers and iron metabolism in women with PMS. However, it failed to show changes indicative of a benefit from curcumin, except for a reduction in hsCRP. It is noteworthy that the baseline hsCRP value was already low (223).

### 5.4 Conclusion

In conclusion, despite the theoretically beneficial effects of curcumin, its conclusive efficacy for PMS symptoms has not been established. While a recent study by Bahrami et al. suggests improvements in cognitive function for women with PMS (224), isolated reports on its effectiveness are insufficient to draw concrete conclusions.

## 6 Alternative treatment

The most common treatment for PMS is pharmacotherapy. However, non-pharmacological methods, such as cognitive-behavioral therapy (CBT), regular aerobic exercise, yoga, vitamin supplementation, and leading a healthy lifestyle, are increasingly recommended as additional options (225).

### 6.1 Cognitive-behavioral therapy

Cognitive-behavioral therapy (CBT) is a psychotherapy that aims to identify negative, disturbing, or destructive thought patterns and develop coping strategies (10) to alleviate associated symptoms, such as depression, stress, and anxiety (226).

Ussher and Perz suggest that couples may benefit more from CBT than individual therapy. Women may feel symptoms such as depression, anger, and irritability during the premenstrual phase (226, 227), which can lead to increased conflict in the relationship with their partner (228, 229). Conversely, women may experience feelings of guilt or other negative thoughts during quiescence. The study involved four 90-minute therapy sessions with a clinical psychologist over 5 months. The focus of the meetings was to challenge these negative thoughts and develop coping strategies. Women were encouraged to engage in self-care and make lifestyle changes, such as exercise and diet. Regardless of the treatment modality, both treatment groups showed improvements in women’s well-being compared to the control group. The study found a sustained reduction in depression, anxiety, and stress over the following three months (226). The results emphasize the significance of receiving understanding and support from loved ones during PMS treatment, as well as the importance of informed education about PMS.

The authors of a separate study demonstrated that CBT can enhance the quality of life for young women with PMS. They highlighted that the knowledge gained during therapy sessions on problem-solving, stress management, and education about a healthy diet can significantly impact positive treatment outcomes (230).

### 6.2 Supplementation

Zinc (Zn) supplementation can provide antioxidant, anti-inflammatory, and antidepressant benefits as a micronutrient (231, 232). Several studies have found that women with PMS have lower levels of zinc (Zn) (233–236). Jafari et al. confirmed that zinc supplementation for 12 weeks reduced the severity of both physical and psychological symptoms of PMS. The study reported an increase in BDNF and TAC levels, which may have contributed to the positive effects of the treatment (232).

Vitamin D plays a crucial role in maintaining calcium homeostasis, sex hormone concentrations, and the normal functioning of neurotransmitters (237–240). In addition, it reduces the production of prostaglandins (241). Furthermore, it has significant effects on the female reproductive system (242). Bahrami et al. demonstrated that high doses of vitamin D (50,000 IU cholecalciferol/week for nine weeks) alleviate PMS symptoms and painful menstruation in adolescent females. The treatment also reduces the incidence of back pain, tendency to cry, and possibly nausea, as well as loss of concentration or lack of energy (243). However, the results of another study contradict this, as it found no significant effect of vitamin D supplementation on PMS symptoms despite administering a dose of 2000 IU every other day for 12 weeks (244).

One study found that calcium supplementation can reduce affective and behavioral symptoms of PMS, such as depression, changes in appetite, and early fatigue (245). Another research confirmed that taking 500mg of calcium daily for two months can reduce mood disturbances related to PMS (246). Earlier studies have also noted a reduction in PMS symptoms (247, 248).

B vitamins play a crucial role in the metabolic processes of many of our body’s systems (19). In a comparative study by Chocano-Bedoya PO et al., it was demonstrated that vitamins B1 and B2 significantly reduce the risk of PMS (249). Abdollahifard et al. found that taking vitamin B1 during the luteal phase reduces both physical and psychological symptoms of PMS (250). Some studies have shown that vitamin B6 can alleviate and reduce the occurrence of PMS symptoms (251–253). B vitamins affect neurotransmitter metabolism through various mechanisms (249). Vitamin B1 is important in the metabolism of GABA precursors, which regulate conductance thought to be crucial in the pathogenesis of PMS (254, 255). Vitamin B2 is essential for the activation of vitamin B6. Vitamin B6 plays a crucial role as a cofactor in the production of serotonin. In contrast, several other studies have not found clear evidence of vitamin B6 effectively modifying PMS symptoms (256–258). However, despite the conflicting results, it is recommended that B vitamins be supplemented as they may help alleviate mild PMS symptoms through the mechanisms described above (259).

Magnesium (Mg) is a cofactor for many enzymes and influences many biochemical reactions. It is responsible for protein synthesis, proper muscle and nerve function, and maintaining blood osmoticity (260). According to Moslehi et al.’s meta-analysis, there is currently no significant relationship between serum or erythrocyte magnesium concentrations.

Additionally, there was no observed vitamin A deficiency in either the luteal or follicular phases. However, it is important to note that the study had a small sample size, consisting of only 10 PMS patients in the study group and 10 women in the control group (261).

### 6.3 Changing to a healthier lifestyle

In addition to the non-pharmacological treatments mentioned above, such as supplementation and physical activity, other factors can influence a healthy lifestyle and potentially improve quality of life.

Research has shown that diet can affect the likelihood of experiencing PMS symptoms. Two studies have confirmed that consuming high amounts of unhealthy foods, such as fast food, soft drinks, processed meat, salt, sugar, sweets, hydrogenated fats, mayonnaise, high-fat dairy products and red meat, can increase the risk of PMS (262, 263). In contrast, consuming meals that are rich in fruits, vegetables, dried fruits, nuts, legumes, garlic, and fish may decrease the likelihood of experiencing PMS (263).

Some studies have indicated that the consumption of caffeine and caffeinated beverages is linked to PMS symptoms, particularly increased breast pain. Therefore, it is recommended to avoid consuming caffeine (264–267). However, several studies have failed to find any correlation (268–270). Therefore, further research is required to determine conclusively whether caffeine has any impact on PMS.

In addition to consuming high-calorie, fatty, sugary, and salty foods, smoking (268, 271–273) and alcohol consumption (274) are also significant risk factors for PMS.

### 6.4 Aerobic exercise and yoga

Physical activity may alleviate some symptoms, such as depression or increased pain tolerance, by increasing the secretion of endorphins and reducing cortisol concentrations (275). Aerobic exercise can also reduce feelings of fatigue, improve concentration, and reduce the intensity of other PMS symptoms. Regular physical activity has been shown to have a regulating effect on prolactin, oestradiol, and progesterone concentrations, as well as an increase in hemoglobin, erythrocytes, and thrombocytes (276).

It is important to note that regular exercise can alleviate PMS symptoms, while occasional physical activity may exacerbate them (277).

Mohebbi Dehnavi et al. confirmed that regular aerobic exercise has a positive effect on PMS symptoms. In their study, 35 female students performed high-intensity aerobic activity for 30 minutes, 3 times a week, for a total of 8 weeks. The completion of it resulted in a reduction in the severity of some physical symptoms of PMS (278).

However, the results of studies on this topic are contradictory. Several of them indicate that aerobic exercises, such as walking, running, or swimming, have a positive impact on PMS symptoms (279–281). Others have found no significant association between physical activity and physical symptoms of PMS (282, 283).

Yoga has been found to have a positive effect on reducing both physical and psychological symptoms of PMS, as evidenced by several studies (284–286). Yoga comprises three key elements: breath control (pranayama), postures (asana), and meditation (dhyana) (287). The breathing techniques employed during yoga have a significant impact on various bodily systems, including the nervous system. They regulate the function of the autonomic nervous system (ANS) and inhibit its sympathetic part. They also reduce heart rate and blood pressure (288, 289). Presumably, this helps relax the mind and body and can relieve feelings of tension, anxiety, or depression (284). Yoga poses involve limb positioning and muscle contraction, which stimulates pressure receptors under the skin. By increasing vagus nerve activity, cortisol production is reduced (290). Furthermore, yoga increases BDNF levels (291), a factor whose increase is one of the resultant effects of SSRI drugs. This effect can be linked both to the activation of the autonomic system during exercise and to the stimulation of prefrontal cortex activity during meditation (292) (293). This can have positive effects on pain, depression, and immune function (284). Meditation, also known as relaxation training or relaxation exercises, involves achieving calmness and inner tranquility by maintaining a continuous state of mindfulness. Furthermore, they stimulate the secretion of melatonin, which enhances the quality of sleep and aids in falling asleep (291).

According to a study conducted by Chang et al., yoga alleviates both physical and psychological symptoms associated with PMS. In a research conducted by Vaghel et al., 65 women performed yoga exercises at home using a 30-minute DVD program, at least three times a week for three menstrual cycles (284).

Both yoga and aerobic exercise were found to have a positive effect on reducing the intensity of pain and other somatic symptoms of PMS. However, the group of women who regularly practiced yoga had better results compared to the group who regularly did aerobic exercise. Yoga may alleviate psychological symptoms of PMS, such as stress and anxiety, due to its focus on breath control and meditation in addition to physical activity, unlike aerobic exercise (294). Yoga can support a focus on feelings, which correlates with some of the components of behavioral-cognitive therapy.

### 6.5 Conclusion

Non-pharmacological treatments can significantly alleviate physical symptoms of PMS. These include supplementation with vitamin B6, vitamin D, zinc, calcium. CBT therapy can positively reduce the severity of PMS symptoms related to the psyche. Regular aerobic exercise and yoga can relieve physical complaints such as headaches, fatigue, cramps, swelling, and breast pain. By using breathing techniques and meditation during yoga, it is possible to reduce feelings of tension, depression, or anxiety. Limiting the intake of unhealthy food, alcohol, and smoking can also reduce the risk of symptoms.

It is worth noting that the treatment methods listed are interrelated and, when used together, can produce positive results. CBT therapy is designed to provide patients with appropriate psychoeducation. Women experiencing PMS can acquire a fundamental understanding of the condition, its causes, and coping mechanisms. Therapy sessions often promote a healthier lifestyle, including regular exercise, and dietary changes, which can alleviate most physical symptoms. Overall, this may improve women’s well-being and quality of life. However, there is a lack of research that considers most non-pharmacological treatments simultaneously.

## 7 Surgical treatment

It is believed that hysterectomy combined with bilateral removal of the ovaries and fallopian tubes (TAH/BSO) can be considered an effective treatment for severe PMS or PMDD. However, it is important to be aware of the consequences it carries - patients are at risk of premature menopause with this form of treatment (295). Ovarian failure after hysterectomy alone is estimated to occur in 15-50% of cases. Due to the risk of the consequences of premature menopause: osteoporosis, premature death, mood disorders, infertility, neurological and cardiovascular diseases, patients are recommended hormone therapy (HRT) (296).

A study by Cronje et al. showed the efficacy of (TAH/BSO) in 47 patients with severe PMS, who continued hormone therapy after surgery. Interestingly, 96% of the women were ‘satisfied’ or ‘very satisfied’ with TAH/BSO, and 93.6% reported complete resolution of cyclic symptoms. The study concluded that TAH/BSO, in combination with HRT, was an extremely effective treatment for PMS (297).

Another invasive treatment for PMS is thermal ablation of the endometrium. A study that tested the efficacy of this form of therapy involved 36 patients reporting heavy periods and PMS symptoms. The average age of the women undergoing treatment was 41.4 years and the average body mass index was 26.7. Most of them, as many as 75 percent, had not undergone effective hormone therapy. Virtually all the women, 97%, reported improvement in PMS after endometrial ablation, both in terms of the severity of symptoms and in terms of the symptoms themselves (298). At this point, it should be noted that symptom relief may be related to the cessation of bleeding itself. In particular, a study involving 73 women showed that initially after treatment when patients did not experience monthly bleeding, they did not report PMS symptoms at the same time, whereas when bleeding recurred, PMS-related complaints also appeared (299).

Surgical methods of treating severe PMS or PMDD, are very effective, however, the complications they may entail should not be overlooked. TAH/BSO appears to be the riskiest, and the potential health risks associated with organ removal require the implementation of hormone replacement. It should also be borne in mind that the studies referred to included a small group and that ablation is not a recognized form of treatment for PMS and PMDD and the impression of a reduction in the severity of complaints may have been correlated with the non-occurrence of bleeding.

## 8 Discussion

Non-pharmacological treatments should be considered before introducing medications. However, in cases of severe symptoms, consider them complementary support for pharmacological treatment. Psychoeducation plays a pivotal role at every stage, helping patients prepare for the most challenging days of the cycle. It should cover elements such as diet and physical activity levels, while fostering awareness of the disorder’s complexity. CBT remains a viable option at any stage of the treatment process.

We recommend that pharmacological treatment should be tailored to the individual clinical needs of the patient, taking into account the entire clinical picture. In the case of the predominance of psychological symptoms, SSRI drugs are preferred, while physical symptoms are treated with OC drugs. It is important to personalize the treatment not only based on the patient’s predominant symptoms but also taking into consideration their preferences and individual circumstances. For instance, for patients who often forget to take their medication, SSRIs might be a better option as they are fast-acting in PMS. Similarly, it is crucial to consider the significant side effects of the drug. For example, paroxetine has a strong anticholinergic effect and can cause weight gain, making it necessary to use caution when considering its inclusion in cases of coexisting diabetes or obesity. On the other hand, escitalopram may cause QT prolongation at higher doses, making it better to choose another drug such as sertraline for patients who are in the habit of self-medication and increasing doses. In case a patient chooses venlafaxine, it is important to keep in mind that it may raise blood pressure.

The most effective approach to treatment with SSRIs appears to be their administration during the luteal phase. Continuous administration is feasible, but it should be reserved for severe cases of PMS (e.g., in patients burdened with other psychiatric disorders) due to the higher risk of side effects. Notably, comparable therapeutic effects can be achieved by administering the drug solely during the luteal phase, making non-continuous administration the preferred treatment method. A continuous treatment regimen may also be considered if there is no response to the intermittent method (only in the luteal phase).

Based on the information presented in our study, the optimal selection among SSRIs would be fluoxetine, which can be used at a maximum dose of 20 mg. Scientific reports suggest no significant differences in side effects between 20 mg and 10 mg doses due to the interval in administration. However, we recommend restricting the administration of the maximum dose to the most severe cases. For others, the preferred starting dose is 10 mg, as studies indicate noticeable improvements in patients’ conditions even at low doses. A follow-up visit is recommended after 3 cycles, as this is when response rates are typically high.

In cases where physical symptoms and irregular cycles predominate, OC drugs appear to be a preferable choice. Among the EE drugs, drospirenone has been extensively studied, with no demonstrated advantage of other preparations in the same group.

**Figure 4:**
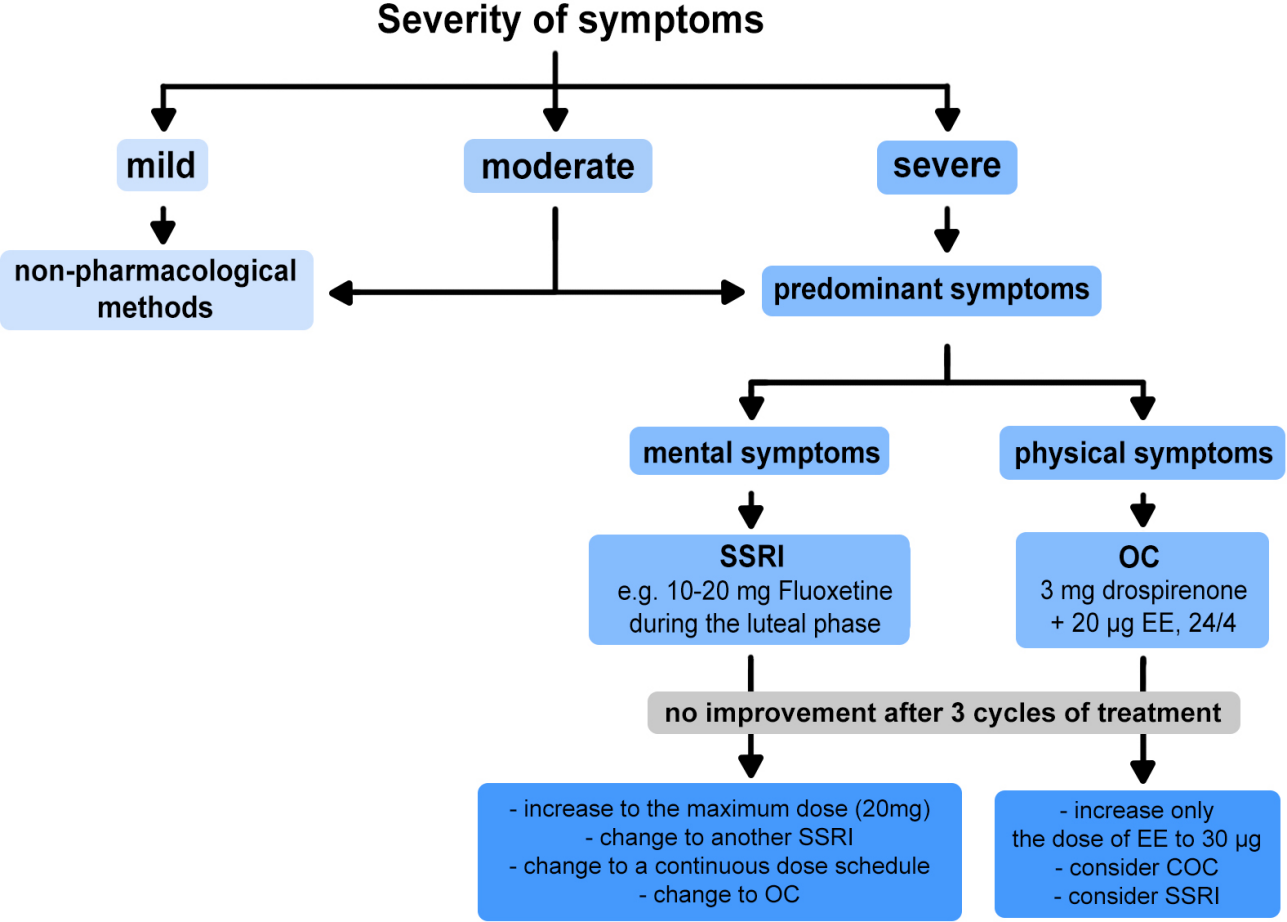
Sample treatment regimen for PMS; e.g. - for example; EE- ethinylestradiol.

We recommend using a dosing schedule of 24/4 instead of 21/7, as supported by the studies we cited. These studies indicate that limiting placebo days has a beneficial effect on treatment. If the placebo regimen proves ineffective in symptom reduction, especially during the placebo phase, transitioning to a COC may be considered (**Figure 4**).

Considering the disorder’s etiology, pain medications may offer a viable option. Additionally, research on neurosteroids suggests that they can be employed to alleviate PMS symptoms by inhibiting the action or synthesis of allopregnanolone. The use of isoallopregnanolone appears particularly promising, although further research is needed to conclusively demonstrate its therapeutic effectiveness.

Herbal treatment serves as an alternative to pharmacological drugs; however, due to significant limitations in the methodology of research on herbs, they should not be considered a therapeutic option in more severe cases. Moreover, numerous interactions restrict the possibility of their use, and while VAC or saffron seem to be the most promising, evidence of their efficacy is limited. Due to the widespread popularity of herbal supplements (300), it is advisable to ensure that patients do not concurrently use such preparations, as monotherapy is preferred to avoid potential interactions. When combining herbs with pharmacological drugs, there is a specific concern regarding their impact on hepatic drug metabolism. For example, St. John’s wort (Hypericum perforatum) acts as an inducer of CYP3A4, CYP1A2, and CYP2C9, which may lead to decreased efficacy of several antidepressants, such as paroxetine, sertraline, and fluoxetine, due to increased liver metabolism. On the other hand, the use of Ginkgo biloba, by inhibiting cytochromes, may elevate plasma concentrations of drugs, potentially increasing side effects and exacerbating the antiplatelet effects of SSRI and SNRI drugs (301). Additionally, it is essential to note that patients using saffron are at a higher risk of developing serotonin syndrome when concurrently taking SSRI drugs.

It is essential to note that our review is not systematic, and despite attempts to cover all studies, one should keep in mind significant limitations.

## Author contributions

SM: Conceptualization, Data curation, Methodology, Project administration, Resources, Visualization, Writing – original draft, Writing – review & editing. AO: Data curation, Investigation, Project administration, Resources, Writing – review & editing. XZ: Data curation, Methodology, Resources, Writing – original draft. KI: Data curation, Investigation, Resources, Visualization, Writing – original draft. ZS: Conceptualization, Visualization, Writing – original draft. NW: Funding acquisition, Project administration, Supervision, Visualization, Writing – review & editing.
